# Biological studies on exogenous NO stress to improve the quality of Radix Scutellariae herbs

**DOI:** 10.1371/journal.pone.0339961

**Published:** 2025-12-31

**Authors:** Kai Zhao, Yafei You, Jincai Lu, Ling Cao, Xiangcai Meng

**Affiliations:** 1 Department of Pharmacognosy, Heilongjiang University of Chinese Medicine, Harbin, Heilongjiang, China; 2 Department of Medicinal Plants and Chinese Herbal Medicine Identification, Shenyang Pharmaceutical University, Shenyang, Liaoning, China; Kyung Hee University, KOREA, REPUBLIC OF

## Abstract

Radix Scutellariae, a crucial botanical drug widely used in Asian countries, is derived from the roots of *Scutellaria baicalensis* Georgi and exhibits diverse pharmacological activities, including antipyretic, analgesic, anti-inflammatory, antibacterial, antiviral, sedative, hypotensive, hypolipidemic, hepatoprotective, and choleretic effects. Owing to excessive market demand, the wild resources are insufficient, and cultivated Radix Scutellariae has thus become the primary source; however, this shift has resulted in a decline in medicinal quality. To address this quality issue, the present study proposes the hypothesis that exogenous nitric oxide (NO) enhances secondary metabolism in fresh roots of *S. baicalensis* Georgi via reactive oxygen species (ROS)-mediated pathways, thereby improving its medicinal quality. Fresh roots of *S. baicalensis* Georgi were treated with sodium nitroprusside (SNP) at concentrations of 0.0, 7.5, and 20 mmol/L to induce ROS bursts. At the optimal concentration of 20 mmol/L SNP, the levels of superoxide anion radicals (O_2_·⁻), hydrogen peroxide (H_2_O_2_), and malondialdehyde (MDA) increased by 95.1%, 134.5%, and 211.5%, respectively (*P* < 0.01). Correspondingly, the activities of antioxidant enzymes—including superoxide dismutase (SOD), catalase (CAT), and peroxidase (POD)—rose by 87.2%, 47.5%, and 59.4%, respectively (*P* < 0.01). The activity of phenylalanine ammonia-lyase (PAL), a key enzyme in flavonoid biosynthesis, increased by 70% (*P* < 0.01). While the contents of major secondary metabolites (baicalin and wogonoside) remained stable, the levels of their high-activity derivatives (baicalein and wogonin) increased dramatically by 1603.2% and 1256.1%, respectively (*P* < 0.01). Pharmacological assays revealed that 20 mmol/L SNP treatment enhanced pharmacological activities: the inhibition rate of body temperature elevation in rats, the inhibition rate of writhing response in mice, and the inhibition rate of auricular swelling in mice increased by 11.7%, 14.9%, and 27.4% (*P* < 0.01), respectively. This treatment also reduced serum interleukin-6 (IL-6) and tumor necrosis factor-*α* (TNF-*α*) levels in mice by 13.5% and 16.8% (*P* < 0.01), respectively. Collectively, this study confirms that exogenous NO induces ROS bursts in fresh *S. baicalensis* Georgi roots and improves the quality of cultivated Radix Scutellariae via ROS-mediated enhancement of secondary metabolism, and clarifies the NO-ROS-secondary metabolism regulatory axis, providing valuable insights for quality improvement strategies of other medicinal plants.

## Introduction

Radix Scutellariae as a herbal medicine derived from the dried roots of *S. baicalensis* Georgi (Lamiaceae) is predominantly produced in Heilongjiang, Jilin, Liaoning, Gansu, Shanxi, Shaanxi, Hebei, and Inner Mongolia in China [1]. Its medicinal effects are primarily attributed to flavonoids, including baicalin, wogonoside, baicalein, and wogonin, which exhibit a range of biological activities such as antipyretic, analgesic, anti-inflammatory, antibacterial, antiviral, sedative, hypotensive, hypolipidemic, hepatoprotective, cholagogic, antiallergic, and smooth muscle spasmolytic properties [2,3]. Radix Scutellariae is in high demand across China and other Asian countries. Owing to its potent antiviral activity, it has played a crucial role in the prevention and treatment of the common cold and COVID-19 in China [4]. The market demand for Radix Scutellariae remains consistently high, with an annual consumption of 20,000 tons. Given that wild resources are insufficient to meet this demand, cultivated Radix Scutellariae has emerged as the primary source; however, this transition has also led to a decline in quality, primarily attributable to lower content of major chemical components and weaker pharmacological activity [5,6]. Therefore, improving the quality of cultivated Radix Scutellariae has emerged as a pressing concern.

ROS are natural substances produced during plant growth, including O_2_· ⁻ , H_2_O_2_, hydroxyl radical (·OH), singlet oxygen (^1^O_2_), and hydroperoxyl radical, among others [7]. Under environmental stress, a large amount of ROS can be generated [8,9]. Since plants cannot move to avoid adverse environments, their internal ROS levels will inevitably increase once plants are exposed to environmental stress [10]. High concentrations of ROS tend to induce cascading oxidative damage, causing changes in molecular structure, disruption of biological membranes, DNA damage, peptide chain breakage, and protein cross-linking, ultimately interfering with metabolism and triggering cell death [11,12]. However, the fundamental reason why plants can survive under severe environmental stress lies in their evolution of secondary metabolic pathways to scavenge excessive ROS [13,14]. Notably, these stress-induced secondary metabolites are typically the active components of medicinal plants [15]. Therefore, ROS outbursts triggered by environmental stress are an important fundamental factor that promotes the biosynthesis of secondary metabolites and thereby improves the quality of medicinal materials.

NO is a charge-neutral, highly hydrophobic small-molecule reactive nitrogen species with strong lipophilicity and biofilm penetration ability, enabling free diffusion within and between cells [16,17]. At low concentrations, NO acts as an antioxidant to directly scavenge ROS and indirectly counteract ROS by regulating antioxidant enzyme activities, thereby maintaining cellular redox balance [18,19]. Research has shown that an appropriate concentration of NO induces moderate ROS accumulation in plants, which in turn helps activate plant secondary metabolic pathways and promote the accumulation of secondary metabolites [20–22]. Based on this, the present study uses SNP as an NO donor to induce physiological states in *S. baicalensis* Georgi under environmental stress, explores the effects of exogenous NO on flavonoids, the main pharmacodynamic components of Radix Scutellariae, elucidates the formation mechanism of herb quality, and provides a new approach for improving herb quality.

## Materials and methods

### Plant materials

Three-year-old fresh roots of *S. baicalensis* Georgi, identified by Professor Xiangcai Meng of Heilongjiang University of Traditional Chinese Medicine, were collected on October 4, 2021, from the Radix Scutellariae herb production base in Gannan County, Qiqihar City, Heilongjiang Province, China, where the plants were grown with a row spacing of 20 cm and a plant spacing of about 15 cm. Fifteen kilograms of fresh roots were selected for the study.

### Instruments

TGL-16LM Benchtop High-Speed Freezing Centrifuge (Hunan Xingke Scientific Instrument Co., Ltd., Changsha, China); HH-6 Constant Temperature Water Bath (Gongyi Yuhua Instrument Co., Ltd., Gongyi, China); 752 UV-visible Spectrophotometer (Shanghai Essence Science and Technology Instrument Co., Ltd., Shanghai, China); Thermo Enzyme Labeling Instrument (Thermo Fisher Scientific, Waltham, U.S.A.); MS3 Spinning Vortex Mixer (IKA, Staufen, Germany); METTLER TOLEDO AG135 0.1 mg Analytical Balance (Mettler Toledo, Zurich, Switzerland); DHG-9015A Blast Drying Oven (Shanghai Yiheng Scientific Instrument Co., Ltd., Shanghai, China); KQ-250DB CNC Ultrasonic Cleaner (Kunshan Ultrasonic Instrument Co., Ltd., Kunshan, China); SHB-Ⅲ Circulating Water Multi-Purpose Vacuum Pump (Zhengzhou Great Wall Science and Industry Trade Co., Ltd., Zhengzhou, China); Agilent 1200 High-Performance Liquid Chromatograph (Agilent Technologies, Santa Clara, U.S.A.); DKZ-3B Water Bath Shaker (Changzhou Xunsheng Instrument Co., Ltd., Changzhou, China); SD40 Ice Machine (Guangzhou Guangkun Electric Appliances Manufacturing Co., Ltd., Guangzhou, China); Julabo TW20 Digital Thermostatic Water Bath (Julabo GmbH, Seelbach, Germany); DKQ-02 Perforator (Baoruiida Experimental Instrument Co., Ltd., Shijiazhuang, China).

### Reagents

Total protein (TP) assay kit, H_2_O_2_ assay kit, MDA assay kit, SOD assay kit, CAT assay kit, POD assay kit, PAL assay kit (Nanjing Jiancheng Bioengineering Research Institute, Nanjing, China); Plant NO Enzyme-Linked Immunosorbent Assay (ELISA) Kit, Plant 1,3-DPG ELISA Kit, Mouse TNF-*α* ELISA Kit, and Mouse IL-6 ELISA Kit (Jiangsu Jingmei Biotechnology Co., Ltd., Yancheng, China); O_2_· ⁻ content assay kit (Beijing Solarbio Technology Co., Ltd., Beijing, China); methanol (analytical grade, Tianjin Fuyu Fine Chemical Co., Ltd., Tianjin, China); phosphoric acid (analytical grade, Tianjin Hengxing Chemical Reagent Manufacturing Co., Ltd., Tianjin, China); acetonitrile (HPLC grade, Beijing Dikma Technologies Inc., Beijing, China); SNP (Zhengzhou Pai Ni Chemical Reagent Factory, Zhengzhou, China); dry yeast (Angel Yeast Co., Ltd., Yichang, China); glacial acetic acid (analytical grade, Tianjin Tianli Chemical Reagent Co., Ltd., Tianjin, China); sodium pentobarbital (pharmaceutical grade, Sinopharm Group Industrial Co., Ltd., Langfang, China); xylene (analytical grade, Tianjin Fuyu Fine Chemical Co., Ltd., Tianjin, China); baicalin, wogonoside, baicalein, and wogonin (Chengdu Efa Biotech Co., Ltd., Chengdu, China, with purity of 98%, 98%, 98%, and 99% respectively); saline (Harbin Sanlian Pharmaceutical Co., Ltd., Harbin, China).

### Experimental methods

#### Plant sample handling.

Fresh roots of *S*. *baicalensis* Georgi were divided evenly into three groups according to diameter size and weight, and were uniformly sprayed with SNP solution at concentrations of 0 (CK), 7.5, and 20 mmol/L until the surface droplets were about to drop. The solution was sprayed every 8 h and the treatment lasted for 6 d. The samples were collected on the 0^th^, 1^st^, 2^nd^, 3^rd^, 4^th^, 5^th^, and 6^th^ d, and the sampling time interval was 24 h. The sampling method was performed as follows: (1) Surface sludge and xylem were removed. 0.3 g of each sample collected from more than 5 plants was taken, with a total of 30 samples, and stored in the refrigerator at −80 °C for the determination of the content of O_2_· ⁻ , H_2_O_2_, NO, MDA, and 1,3-DPG and the activities of SOD, CAT, POD, and PAL. (2) Fresh samples of more than 100 g were put in a 55 °C blast drying oven to dry, then crushed, and passed through No. 3 sieve for the determination of baicalin, wogonoside, baicalein, and wogonin contents. The day 0 samples were all obtained from fresh roots without any treatment; thus, they were set as the blank group and tested for all the aforementioned indices in parallel with the experimental groups to observe changes in the indices before and after treatment. For each detection index, triplicate parallel operations were performed for samples in each group.

#### Determination of physiological and biochemical indices.

The homogenized protein content of fresh roots was determined using a TP assay kit; NO content was determined using a plant NO ELISA kit; and O_2_·⁻ and H_2_O_2_ contents were determined using an O_2_· ⁻ detection kit and an H_2_O_2_ assay kit, respectively. Additionally, MDA content was determined using an MDA assay kit based on the TBA method. Furthermore, the activities of antioxidant enzymes including SOD, POD, and CAT were determined using their respective assay kits. Moreover, 1,3-DPG content was determined using a 1,3-DPG ELISA kit, and PAL activity, with PAL being a key enzyme in secondary metabolism, was determined using a PAL assay kit.

#### Determination of secondary metabolite content.

##### Preparation of control solution:

Appropriate amounts of the control products baicalin, wogonoside, baicalein, and wogonin were weighed precisely, and the control solutions with mass concentrations of 1.658, 0.648, 0.957, and 0.528 mg/mL were prepared with methanol solution, respectively.

##### Preparation of test solutions:

1.50 g of Radix Scutellariae powder was weighed and placed in a stoppered conical flask, to which 50.0 mL of methanol solution was precisely added; the flask was stoppered tightly and weighed. Ultrasonic extraction was conducted for 1 h (power 200 W, frequency 40 kHz). The conical flask was cooled down, weighed again, and the lost mass was replenished with methanol; the solution was shaken well, and filtered through a 0.45 μm micropore membrane to obtain the test solutions.

##### Chromatographic conditions:

**Fig 1:**
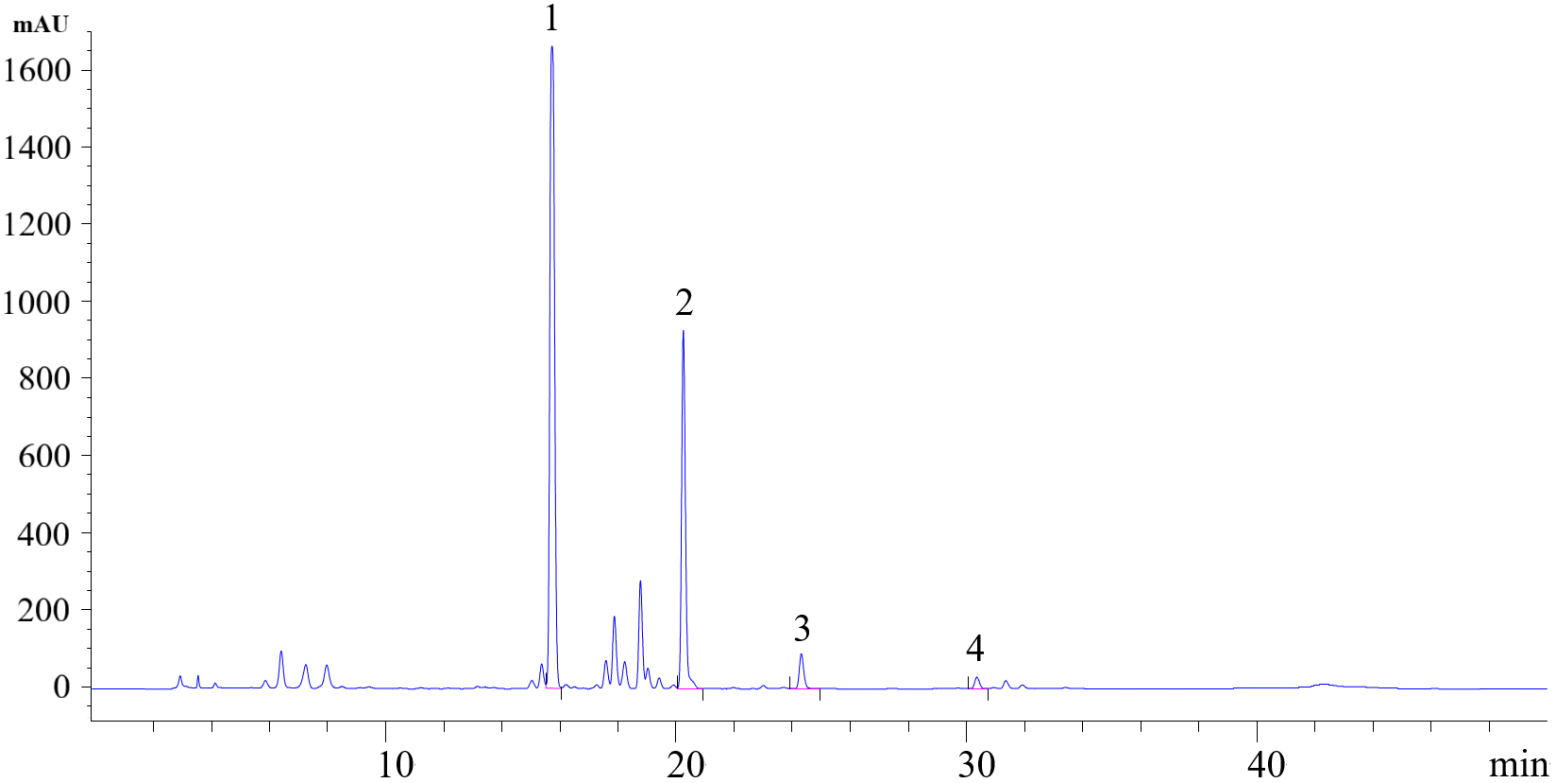
HPLC chromatogram of Radix Scutellariae samples. 1. Baicalin (Retention Time: 15.722 min); 2. Wogonoside (Retention Time: 20.251 min); 3. Baicalein (Retention Time: 24.313 min); 4. Wogonin (Retention Time: 30.352 min).

Column: Diamonsil C_18_ (250 mm × 4.6 mm, 5 μm); mobile phase: acetonitrile (A)**–**0.1% aqueous phosphoric acid (B), gradient elution (0**–**5 min, 20%A; 5**–**35 min, 20%A**–**55%A; 35**–**40 min, 55%A**–**65%A; 40**–**50 min, 65%A**–**20%A); column temperature: 32 °C; injection volume: 10 μL; flow rate: 1 mL/min; detection wavelength: 280 nm. Under these chromatographic conditions, the retention times of the four target compounds were as follows: baicalin (15.722 min), wogonoside (20.251 min), baicalein (24.313 min), and wogonin (30.352 min). The High Performance Liquid Chromatography (HPLC) chromatograms of the experimental samples are shown in Fig 1.

##### Methodological examination:

Linearity: 0.1, 0.2, 0.5, 1.0, 2.0, and 5.0 mL of each control solution under “Preparation of control solution” were taken and diluted to 10 mL with methanol solution to prepare a series of mass concentrations of the control solution, and then the samples were injected repeatedly under the above chromatographic conditions and the peak areas of baicalin, wogonoside, baicalein, and wogonin were recorded. The regression analysis was carried out with the mass concentrations as the horizontal coordinate (*X*) and the peak areas as the vertical coordinate (*Y*), and the regression equations, coefficients of determination *(R*^*2*^) and linear ranges of the four components were calculated, and the results showed that the linear relationship was good within their respective ranges.

Accuracy: Take the mixed solution of the control products within the linear range, repeat the injections six times under the above chromatographic conditions, record the peak areas of baicalin, wogonoside, baicalein, and wogonin, and calculate the Relative Standard Deviations (RSDs) of 0.54%, 0.74%, 0.88%, and 0.87%, respectively, indicating that the instrumental accuracy was good.

Stability: The test solution was obtained according to the method under “Preparation of test solutions”, and the samples were injected and measured at 0, 4, 8, 16, and 24 h under the above chromatographic conditions, and the peak areas of baicalin, wogonoside, baicalein, and wogonin were recorded, and the RSDs were calculated to be 0.49%, 0.73%, 0.96%, and 0.76%, indicating that the stability of the test solution was good within 24 h.

Reproducibility: 6 portions of crude powder of the same group of herbs were taken, each 0.5 g. The test solution was obtained according to the method under “Preparation of test solutions”, and the samples were injected into the instrument for determination under the above chromatographic conditions, and the peak areas of baicalin, wogonoside, baicalein, and wogonin were recorded, and the RSDs were calculated to be 1.01%, 1.47%, 0.91%, and 0.83%, indicating that the method had good reproducibility.

Sample recovery rate: Precisely weigh 0.15 g of crude powder of the same group of herbs, a total of 6 portions, to which an appropriate amount of mixed standard solution was added; the concentrations of baicalin, wogonoside, baicalein, and wogonin were similar to those of the extracts in the tested herbs, respectively. The test solution was obtained according to the method under “Preparation of test solutions”, and the samples were injected under the above chromatographic conditions; the peak areas of baicalin, wogonoside, baicalein, and wogonin were recorded, and the average sample recoveries of baicalin, wogonoside, baicalein, and wogonin were calculated, together with their corresponding RSDs. The results showed that the method had a high sample recovery rate and an accurate determination.

#### Pharmacodynamic verification.

##### Preparation of pharmaceutical solutions:

In this experiment, the quality of Radix Scutellariae on day 6 of the 20 mmol/L SNP group was the best. Powder of Radix Scutellariae at day 0 and from the 20 mmol/L SNP-treated group on day 6 was taken, respectively, added 10 times the amount of water, soaked for 1 h, heated the mixture up to boiling and then kept for 1 h, filtered and then added 8 times the amount of water to the residue and decocted it again for 1 h, and then filtered. After combining the two filtrates, they were concentrated in a water bath to 0.027 g/mL (rat concentration) and 0.039 g/mL (mouse concentration), and stored at −20 °C; these concentrations were determined with reference to the commonly used effective dose of Radix Scutellariae in the 2020 edition of the Chinese Pharmacopoeia and based on the clinical equivalent dose converted using the body surface area of rats and mice to ensure the scientific rationality of concentration selection.

##### Experimental animals and ethical details:

Specific Pathogen-Free (SPF)-grade male Kunming (KM) mice (body weight: 20 ± 2 g) and SPF-grade male Sprague-Dawley (SD) rats (body weight: 200 ± 20 g) were provided by Liaoning Changsheng Biotechnology Co., Ltd. (Benxi, China), with Quality Certificate No. SCXK (Liao)-2020–0001 and Animal Quality Certificate No. 210726231100557273. Animals were housed in a well-ventilated, dry environment (temperature: 20–23 °C; relative humidity: 50–60%; 12 h light/dark cycle) with free access to standard food and water. All animal experiments were approved by the Laboratory Animal Ethics Committee of Heilongjiang University of Chinese Medicine (full approval number: SYXK2023–122922; approved on December 29, 2023) and were strictly conducted in adherence to the National Institutes of Health (NIH) guidelines, ARRIVE guidelines, and the 3Rs principle.

Sample size and total number of animals: A total of 120 animals were used, including 40 SD rats (for antipyretic experiments) and 80 KM mice—40 for analgesic experiments and 40 for anti-inflammatory experiments—with 10 animals per group. This sample size was determined based on previous pharmacodynamic studies of Radix Scutellariae and statistical power analysis, ensuring sufficient statistical validity while minimizing unnecessary usage.

Anesthesia and euthanasia protocols: For mouse euthanasia, prior to the procedure, mice were anesthetized via intraperitoneal (IP) injection of pharmaceutical-grade sodium pentobarbital (1% w/v in sterile 0.9% saline) at 50 mg/kg; deep anesthesia was confirmed by loss of righting reflex, absence of withdrawal response to a 3–5-second toe pinch, and extinction of the corneal reflex. Euthanasia was performed via a lethal IP dose of sodium pentobarbital (150–200 mg/kg) to induce irreversible cardiopulmonary arrest, followed by cervical dislocation to confirm death. For rat euthanasia, prior to the procedure, rats were anesthetized via IP injection of sodium pentobarbital (1% w/v in sterile 0.9% saline) at 60–80 mg/kg; euthanasia was performed with a lethal dose of sodium pentobarbital (200–300 mg/kg), followed by cervical dislocation to confirm death.

##### Antipyretic effect:

The rats were acclimatized to the laboratory environment for 5 days. Anal temperatures were measured three times, 1 h apart, 1 day before the experiment, and the average value was taken as the normal body temperature; rats with body temperature changes of more than 1°C were removed, and the remaining rats were randomly divided into blank, model, untreated Radix Scutellariae, and SNP-treated Radix Scutellariae groups, with 10 rats in each group. The untreated Radix Scutellariae and the SNP-treated Radix Scutellariae groups were administered 10 mL/kg (corresponding to an administered dose of approximately 0.27 g/kg) by gavage at 9:00 a.m. every day, and the blank group and the model group were administered equal doses of saline by gavage for 7 consecutive days. Except for the blank group, the other groups were subcutaneously injected with 20 mL/kg of 15% dry yeast saline suspension (used to establish a rat fever model) into the back at 3 h after the last administration, and the anal temperature was measured at 5 h after the injection. After the anal temperature measurement was completed, the rats were euthanized.

Difference in temperature increase (ΔT) = Temperature after 5 h – Basal body temperature
Temperature suppression rate = (A_1_ - A_2_)/ A_1_ × 100%
A_1_: Temperature elevation of the model group
A_2_: Temperature elevation of the Radix Scutellariae groups

##### Analgesic effect

Forty mice were randomly divided into blank, model, untreated Radix Scutellariae and SNP-treated Radix Scutellariae groups, with 10 mice in each group. The untreated Radix Scutellariae and the SNP-treated Radix Scutellariae groups were administered 10 mL/kg (corresponding to an administered dose of approximately 0.39 g/kg) of the drug solution by gavage at 9:00 a.m. every day, and the blank group and the model group were administered equal doses of saline by gavage for 7 consecutive days, and 0.2 mL of acetic acid with the concentration of 0.6% was intraperitoneally injected at 3 h after the last administration, and the twisting number of the mice was observed in 15 min after the injection and the twisting inhibition rate was calculated. After the 15-minute observation period and calculation of the twisting inhibition rate, the mice were euthanized.

Twisting inhibition rate = (A_1_ - A_2_)/ A_1_ × 100%
A_1_: number of twistings in the model group
A_2_: number of twistings in the Radix Scutellariae groups

##### Anti-inflammatory effects

Forty mice were randomly divided into blank, model, untreated Radix Scutellariae and SNP-treated Radix Scutellariae groups, with 10 mice in each group. The untreated Radix Scutellariae and the SNP-treated Radix Scutellariae groups were administered 10 mL/kg (corresponding to an administered dose of approximately 0.39 g/kg) of the drug solution by gavage at 9:00 a.m. every day, and the blank group and the model group were administered equal doses of saline by gavage for 7 consecutive days. After gavage administration for 3 h on the last day, 50 μL of xylene was applied to both sides of the right ear to cause inflammation, and the left ear was used as a control. Thirty minutes after inflammation induction, the mice were euthanized following the aforementioned protocol to minimize suffering. Ear slices were harvested from the same anatomical location of both the right and left ears using a 6-mm-diameter perforator. These slices were immediately weighed on a precision balance (accuracy: 0.1 mg). The degree of ear swelling was represented by the mass difference between the left and right ear slices, and the swelling inhibition rate was calculated accordingly. Following this, blood was gently collected from the eyeballs of the euthanized mice. The blood samples were allowed to stand at room temperature for 0.5 h; then centrifuged at 3000 r/min for 10 min. The resulting serum was used to determine the levels of TNF-*α* and IL-6.

Swelling inhibition rate = (A_1_ - A_2_)/ A_1_ × 100%
A_1_: Swelling degree of the model group
A_2_: Swelling degree of the Radix Scutellariae groups

#### Data processing methods.

Microsoft Excel 2021 (Microsoft, Redmond, WA, USA) was used to sort the original data. Prism 8 (GraphPad Software, La Jolla, CA, USA) was used for graphic illustration. All data were expressed as mean ± standard deviation (Mean ± S.D.). IBM SPSS Statistics 27.0 (IBM, Armonk, NY, USA) was used for statistical analysis. The normality of data distribution was verified using the Shapiro-Wilk test, and the homogeneity of variances was assessed using Levene’s test. An independent-samples t-test was applied for two-group comparisons, while one-way analysis of variance (ANOVA) followed by Dunnett’s post-hoc test was used for multi-group comparisons. Differences were considered statistically significant at *p < 0.05 or **p < 0.01.

## Results

### ROS content

**Fig 2:**
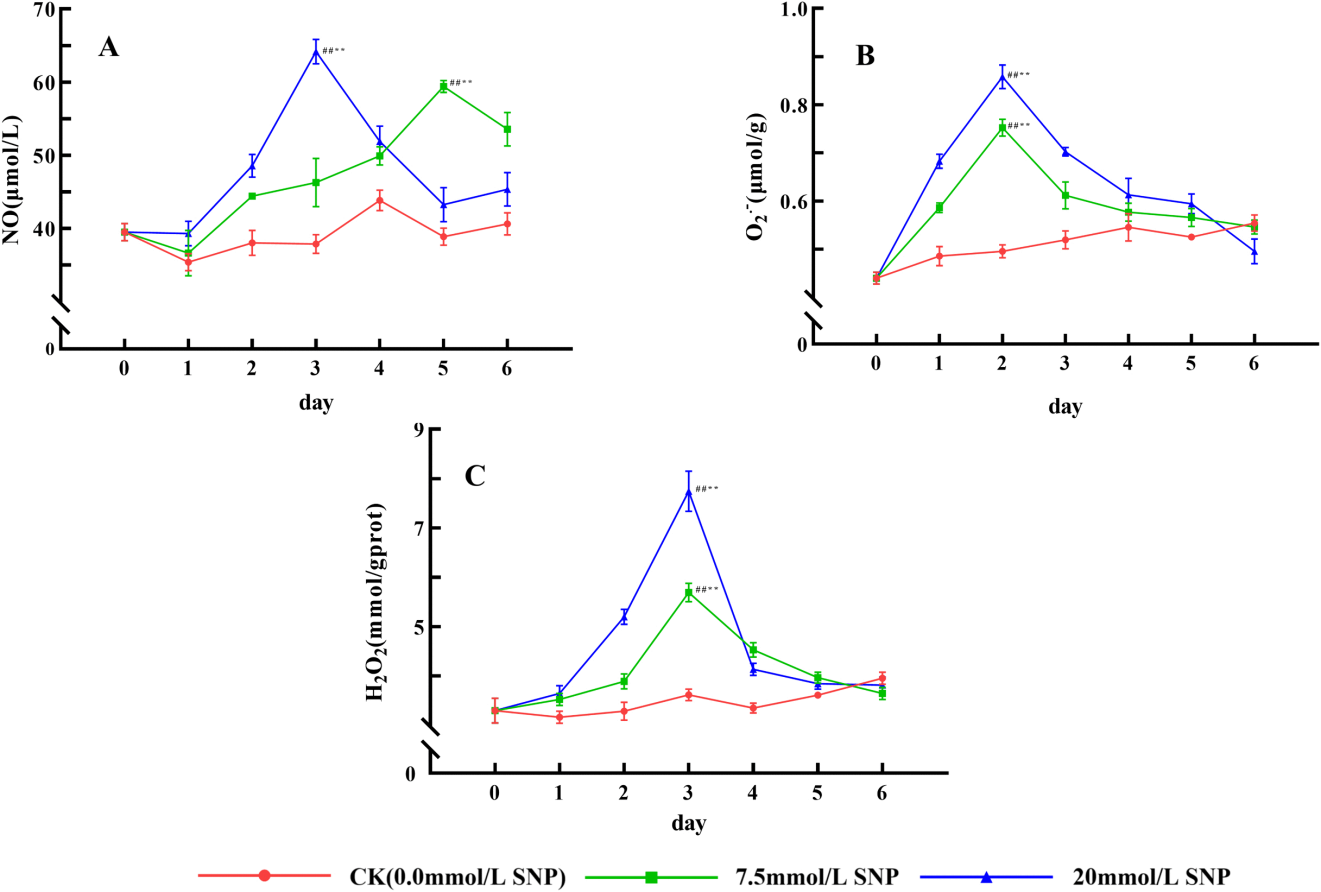
Effect of SNP on ROS contents in *S*. *baicalensis* Georgi roots. A: Effect of SNP on NO contents in *S*. *baicalensis* Georgi roots; B: Effect of SNP on O_2_· ⁻ contents in *S*. *baicalensis* Georgi roots; C: Effect of SNP on H_2_O_2_ contents in *S*. *baicalensis* Georgi roots. Data are presented as $\overset{―}{𝐱}\pm s$ (*n* = 3, error bars: SD). Significance: Compared with day 0 (#: *P* < 0.05, ##: *P* < 0.01); Compared with the CK group (*: *P* < 0.05, **: *P* < 0.01). Groups: CK (0.0 mmol/L SNP, red), 7.5 mmol/L SNP (green), 20 mmol/L SNP (blue). NO contents: μmol/L; O_2_· ⁻ contents: μmol/g; H_2_O_2_ contents: mmol/gprot.

ROS contents of each SNP treatment group generally showed an increasing followed by a decreasing trend. Compared with day 0 (39.5 μmol/L), NO contents in the 20 mmol/L group peaked on day 3 (64.2 μmol/L), increasing by 62.5% (*P* < 0.01); the 7.5 mmol/L group peaked on day 5 (59.4 μmol/L), increasing by 50.4% (*P* < 0.01) (Fig 2A). O_2_· ⁻ contents in the 7.5 and 20 mmol/L groups peaked on day 2 (0.752 and 0.858 μmol/g), increasing by 71.0% (*P* < 0.01) and 95.1% (*P* < 0.01). Compared with day 0 (3.3 mmol/gprot) (Fig 2B), H_2_O_2_ contents in the 7.5 and 20 mmol/L groups peaked on day 3 (5.69 and 7.74 mmol/gprot), increasing by 72.4% (*P* < 0.01) and 134.5% (*P* < 0.01) (Fig 2C). During the treatment period, the ROS contents of the SNP-treated groups were all significantly higher than those of the CK group, indicating that SNP could induce ROS production.

### MDA contents

**Fig 3:**
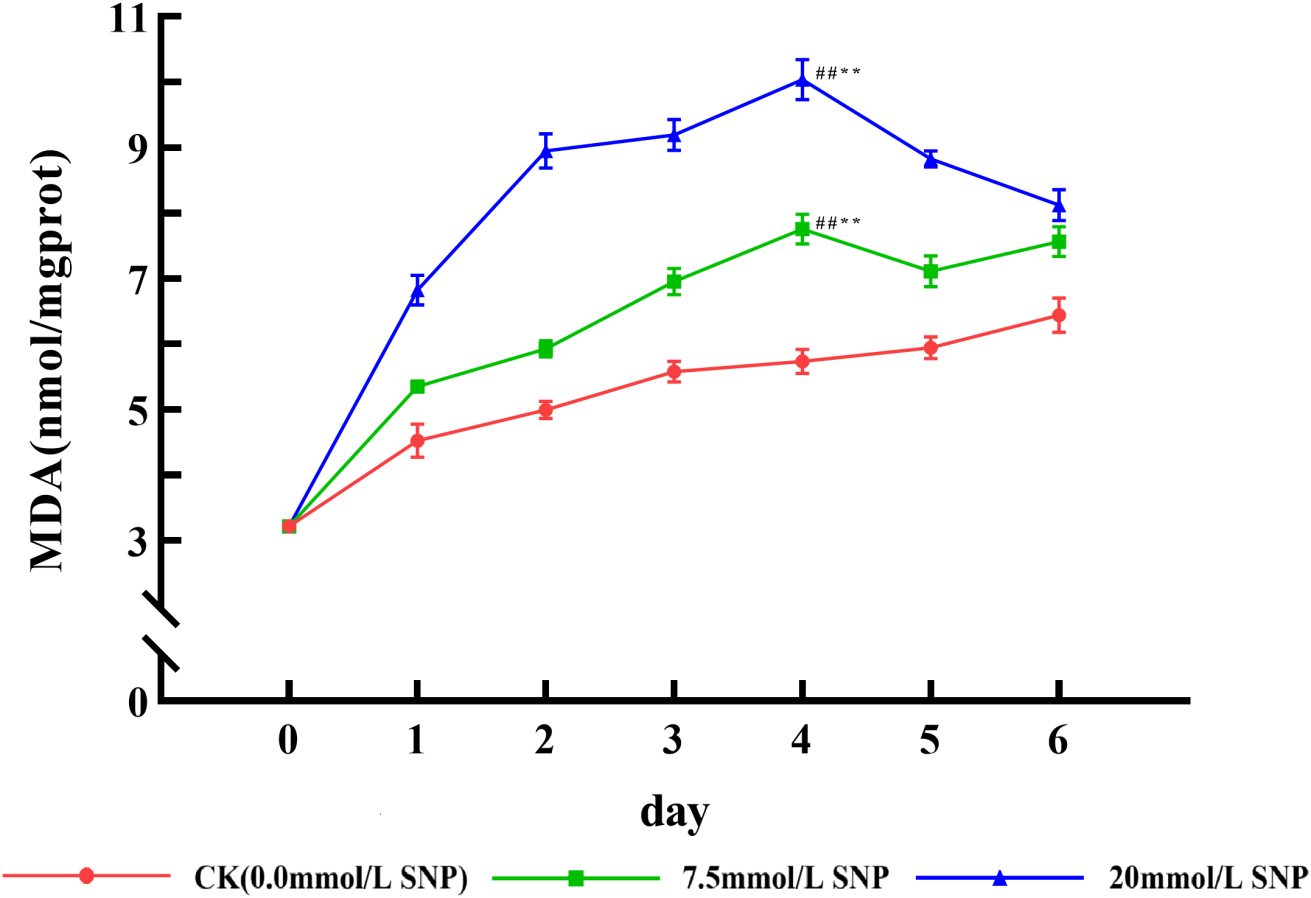
Effect of SNP on MDA contents in *S*. *baicalensis* Georgi roots. Data are presented as $\overset{―}{𝐱}\pm s$ (*n* = 3, error bars: SD). Significance: Compared with day 0 (#: *P* < 0.05, ##: *P* < 0.01); Compared with the CK group (*: *P* < 0.05, **: *P* < 0.01). Groups: CK (0.0 mmol/L SNP, red), 7.5 mmol/L SNP (green), 20 mmol/L SNP (blue). MDA contents: nmol/mgprot.

MDA contents showed an increasing trend in all groups, with the CK group increasing but remaining significantly lower than the SNP-treated groups. Compared with day 0 (3.22 nmol/mgprot), MDA contents in the 7.5 and 20 mmol/L SNP groups peaked on day 4 (7.75 and 10.03 nmol/mgprot), increasing by 140.7% (*P* < 0.01) and 211.5% (*P* < 0.01) (Fig 3). The increase in MDA was positively correlated with the exogenous NO concentration, indicating that SNP caused greater cellular damage and that NO could induce physiological responses in *S. baicalensis* Georgi under environmental stress.

### Antioxidant enzyme activities

**Fig 4:**
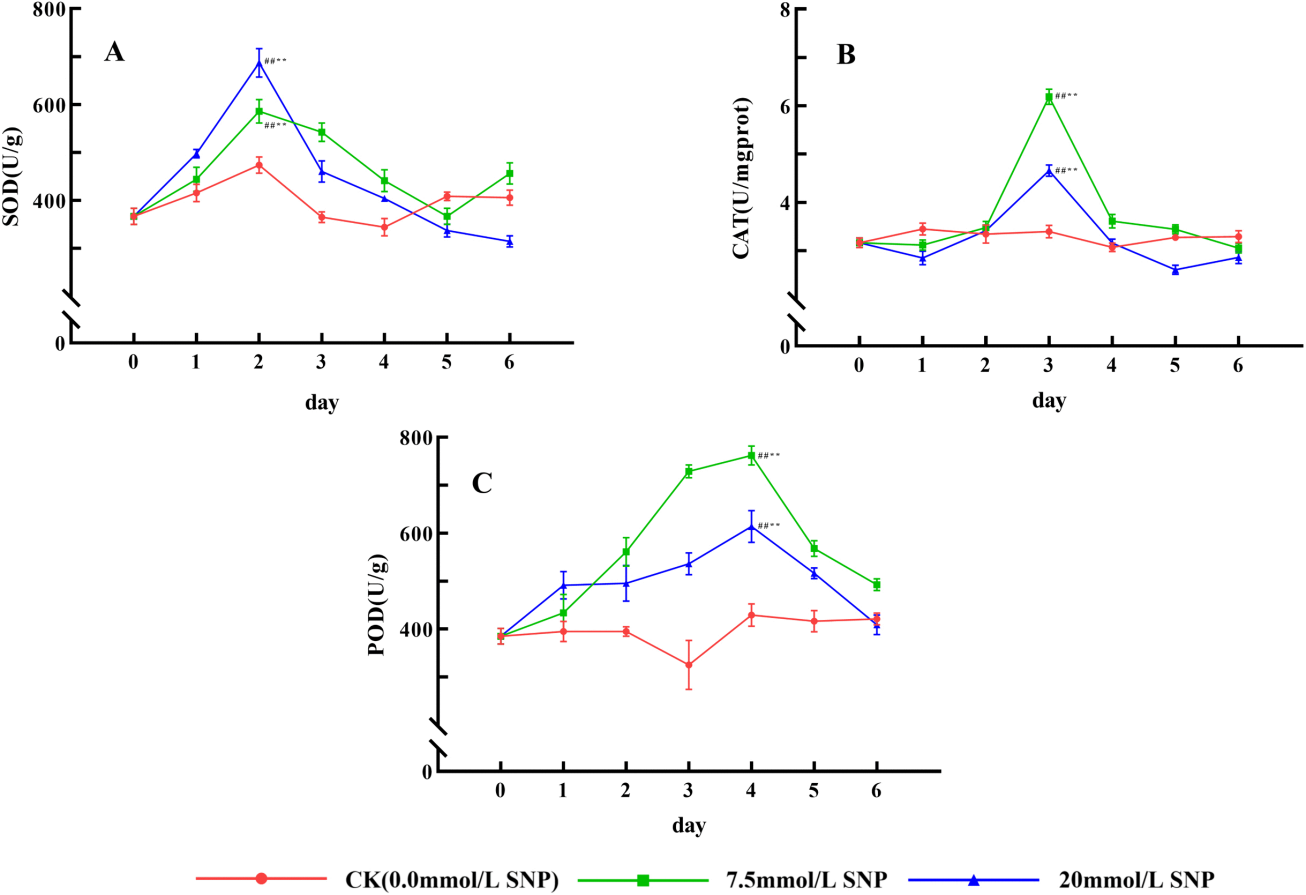
Effect of SNP on antioxidant enzyme activities in *S*. *baicalensis* Georgi roots. A: Effect of SNP on SOD activities in *S. baicalensis* Georgi roots; B: Effect of SNP on CAT activities in *S. baicalensis* Georgi roots; C: Effect of SNP on POD activities in *S. baicalensis* Georgi roots. Data are presented as $\overset{―}{𝐱}\pm s$ (*n* = 3, error bars: SD). Significance: Compared with day 0 (#: *P* < 0.05, ##: *P* < 0.01); Compared with the CK group (*: *P* < 0.05, **: *P* < 0.01). Groups: CK (0.0 mmol/L SNP, red), 7.5 mmol/L SNP (green), 20 mmol/L SNP (blue). SOD activities: U/g; CAT activities: U/mgprot; POD activities: U/g.

Antioxidant enzyme activities of all SNP treatment groups showed an overall trend of increasing and then decreasing. Compared with day 0 (366.9 U/g), SOD activities in the 7.5 and 20 mmol/L groups peaked on day 2 (585.8 and 687.0 U/g), increasing by 59.6% (*P* < 0.01) and 87.2% (*P* < 0.01) (Fig 4A). Compared with day 0 (3.16 U/mgprot), CAT activities in the 7.5 and 20 mmol/L groups peaked on day 3 (6.18 and 4.66 U/mgprot), increasing by 95.6% (*P* < 0.01) and 47.5% (*P* < 0.01) (Fig 4B). Compared with day 0 (385.2 U/g), POD activities in the 7.5 and 20 mmol/L groups peaked on day 4 (762.2 and 614.1 U/g), increasing by 97.9% (*P* < 0.01) and 59.4% (*P* < 0.01) (Fig 4C). The antioxidant enzyme activities of all the above SNP-treated groups were significantly higher than those of the CK group from day 2–4, whereas there was little change in the CK group, indicating that the antioxidant enzymes play an important role in defense against ecological stresses.

### 1,3-DPG content

**Fig 5:**
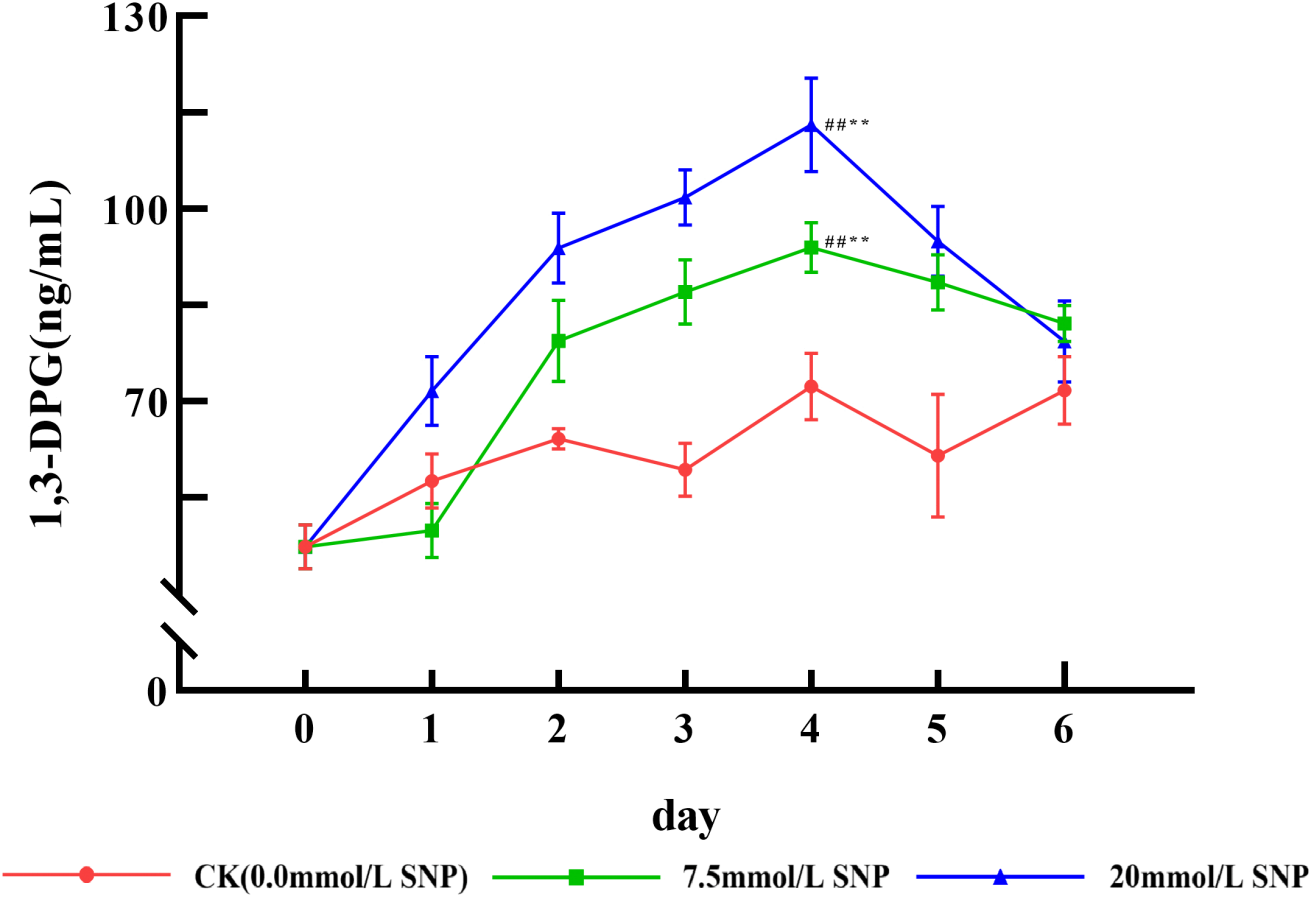
Effect of SNP on 1,3-DPG contents in *S. baicalensis* Georgi roots. Data are presented as $\overset{―}{𝐱}\pm s$ (*n* = 3, error bars: SD). Significance: Compared with day 0 (#: *P* < 0.05, ##: *P* < 0.01); Compared with the CK group (*: *P* < 0.05, **: *P* < 0.01). Groups: CK (0.0 mmol/L SNP, red), 7.5 mmol/L SNP (green), 20 mmol/L SNP (blue). 1,3-DPG contents: ng/mL.

1,3-DPG contents of each SNP treatment group showed an increasing and then decreasing trend. Compared with day 0 (47.3 ng/mL), 1,3-DPG contents in the 7.5 and 20 mmol/L groups peaked on day 4 (94.0 and 113.1 ng/mL), increasing by 98.7% (*P* < 0.01) and 139.1% (*P* < 0.01) (Fig 5).

### Secondary metabolism-related enzymes activities

**Fig 6:**
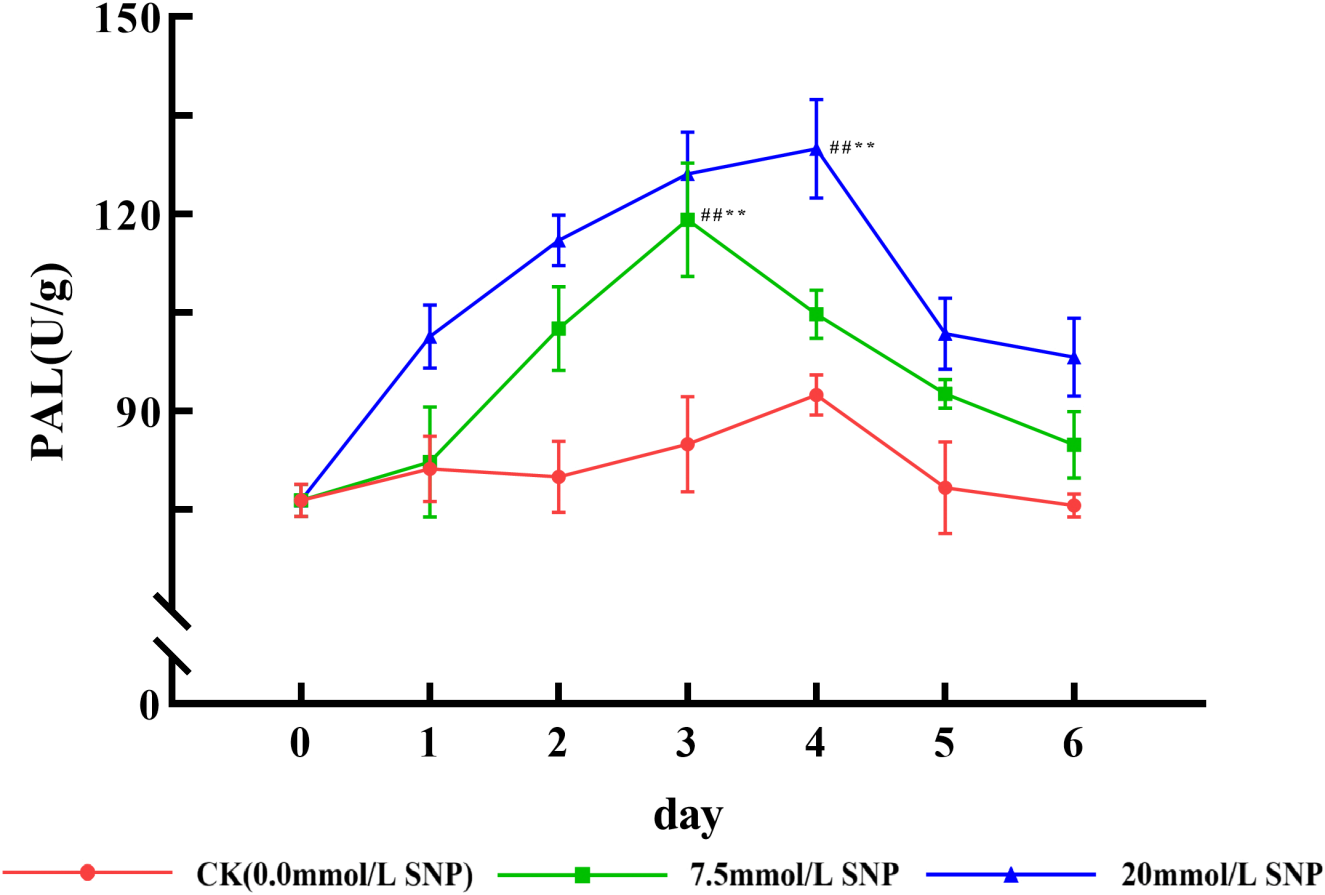
Effect of SNP on PAL activities in *S*. *baicalensis* Georgi roots. Data are presented as $\overset{―}{𝐱}\pm s$ (*n* = 3, error bars: SD). Significance: Compared with day 0 (#: *P* < 0.05, ##: *P* < 0.01); Compared with the CK group (*: *P* < 0.05, **: *P* < 0.01). Groups: CK (0.0 mmol/L SNP, red), 7.5 mmol/L SNP (green), 20 mmol/L SNP (blue). PAL activities: U/g.

PAL activities in all SNP groups showed an increasing and then decreasing trend. Compared to day 0 (76.4 U/g), PAL activities in the 7.5 mmol/L group peaked on day 3 (119.1 U/g), increasing by 55.9% (*P* < 0.01), and PAL activities in the 20 mmol/L group peaked on day 4 (129.9 U/g), increasing by 70.0% (*P* < 0.01) (Fig 6).

### Secondary metabolite content

**Fig 7:**
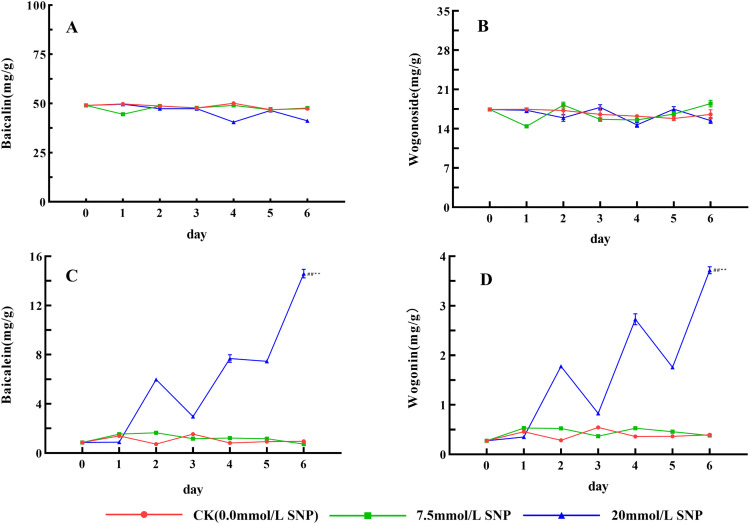
Effect of SNP on major secondary metabolites in *S*. *baicalensis* Georgi roots. A: Effect of SNP on baicalin contents in *S. baicalensis* Georgi roots; B: Effect of SNP on wogonoside contents in *S. baicalensis* Georgi roots; C: Effect of SNP on baicalein contents in *S. baicalensis* Georgi roots; D: Effect of SNP on wogonin contents in *S. baicalensis* Georgi roots. Data are presented as $\overset{―}{𝐱}\pm s$ (*n* = 3, error bars: SD). Significance: Compared with day 0 (#: *P* < 0.05, ##: *P* < 0.01); Compared with the CK group (*: *P* < 0.05, **: *P* < 0.01). Groups: CK (0.0 mmol/L SNP, red), 7.5 mmol/L SNP (green), 20 mmol/L SNP (blue). Baicalin: mg/g; wogonoside: mg/g; baicalein: mg/g; wogonin: mg/g.

There was no obvious changing trend for baicalin and wogonoside contents in all groups (Fig 7A and 7B). However, baicalein and wogonin contents in the 20 mmol/L group showed a rapid and significant increase, compared to day 0 (0.857 and 0.274 mg/g), peaking on day 6 (14.6 and 3.72 mg/g), with increases of 1603.2% (*P* < 0.01) and 1256.1% (*P* < 0.01). In contrast, those in the CK and 7.5 mmol/L groups showed no significant fluctuation during the SNP treatment, with no obvious peak observed (Fig 7C and 7D).

### Pharmacodynamic performance

**Fig 8:**
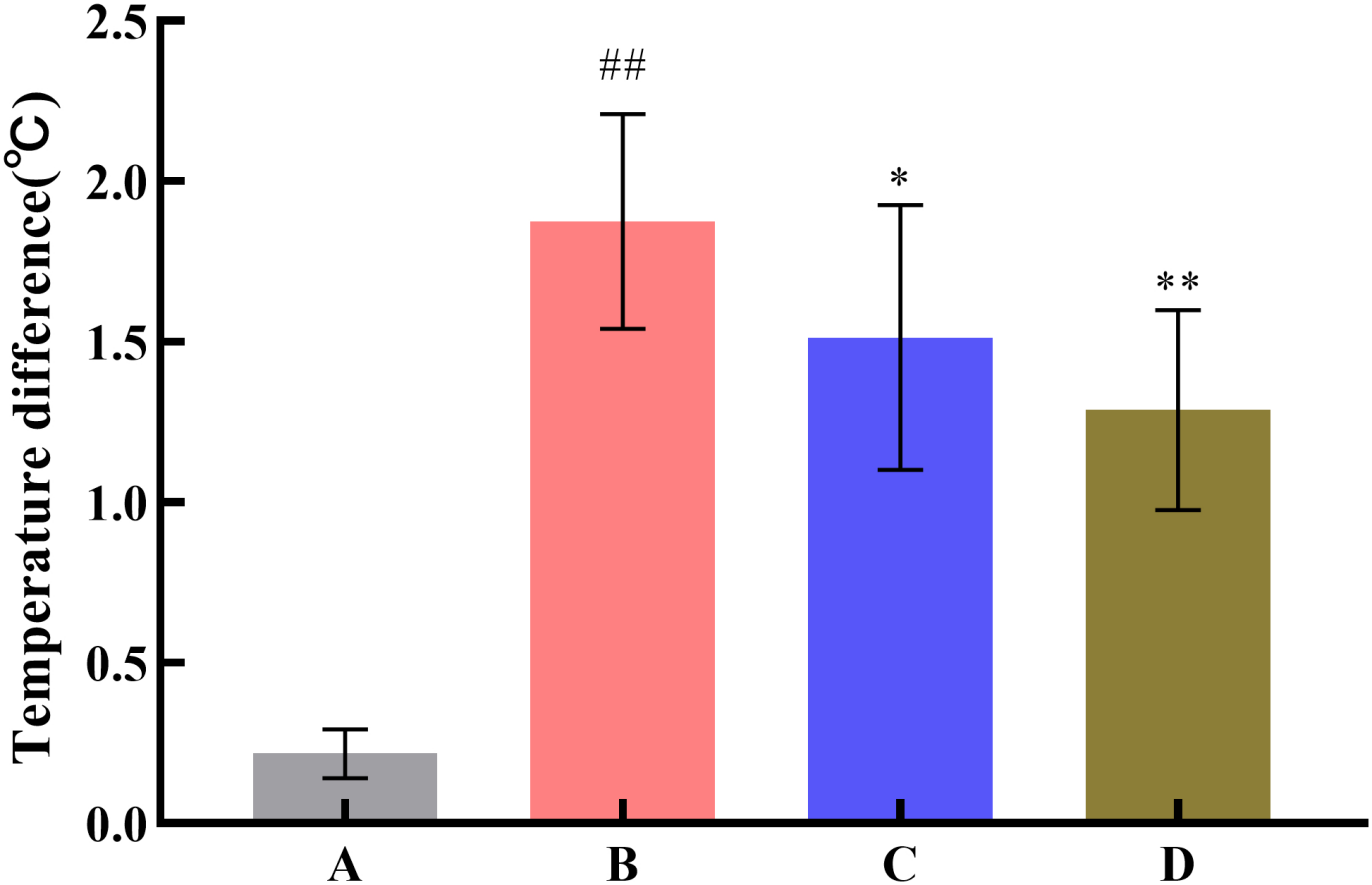
Effect of Radix Scutellariae on body temperature of rats. Data are presented as *x̅* ± *s* (*n* = 10, error bars: SD). Significance: Compared with blank group (^#^: *P* < 0.05, ^##^: *P* < 0.01); Compared with model group (*: *P* < 0.05, **: *P* < 0.01). Groups: A (Blank Group), B (Model Group), C (untreated Radix Scutellariae Group), D (SNP-treated Radix Scutellariae Group). Temperature difference: °C.

**Fig 9:**
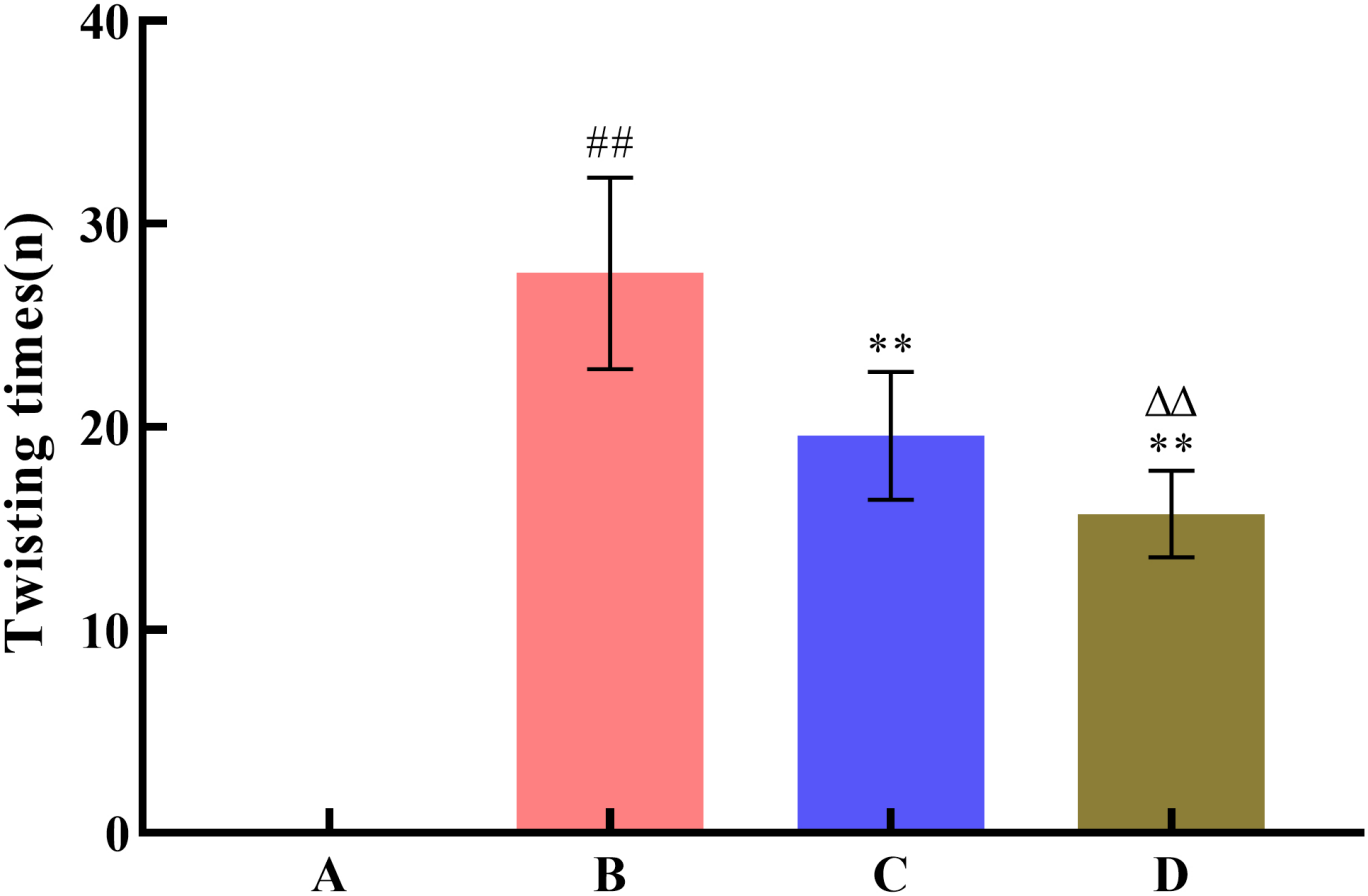
Effect of Radix Scutellariae on pain threshold in mice. Data are presented as *x̅* ± *s* (*n* = 10, error bars: SD). Significance: Compared with blank group (^#^: *P* < 0.05, ^##^: *P* < 0.01); Compared with model group (*: *P* < 0.05, **: *P* < 0.01); Compared with untreated Radix Scutellariae group (^Δ^: *P* < 0.05, ^ΔΔ^: *P* < 0.01). Groups: A (Blank Group), B (Model Group), C (untreated Radix Scutellariae Group), D (SNP-treated Radix Scutellariae Group). Twisting times: n.

**Fig 10:**
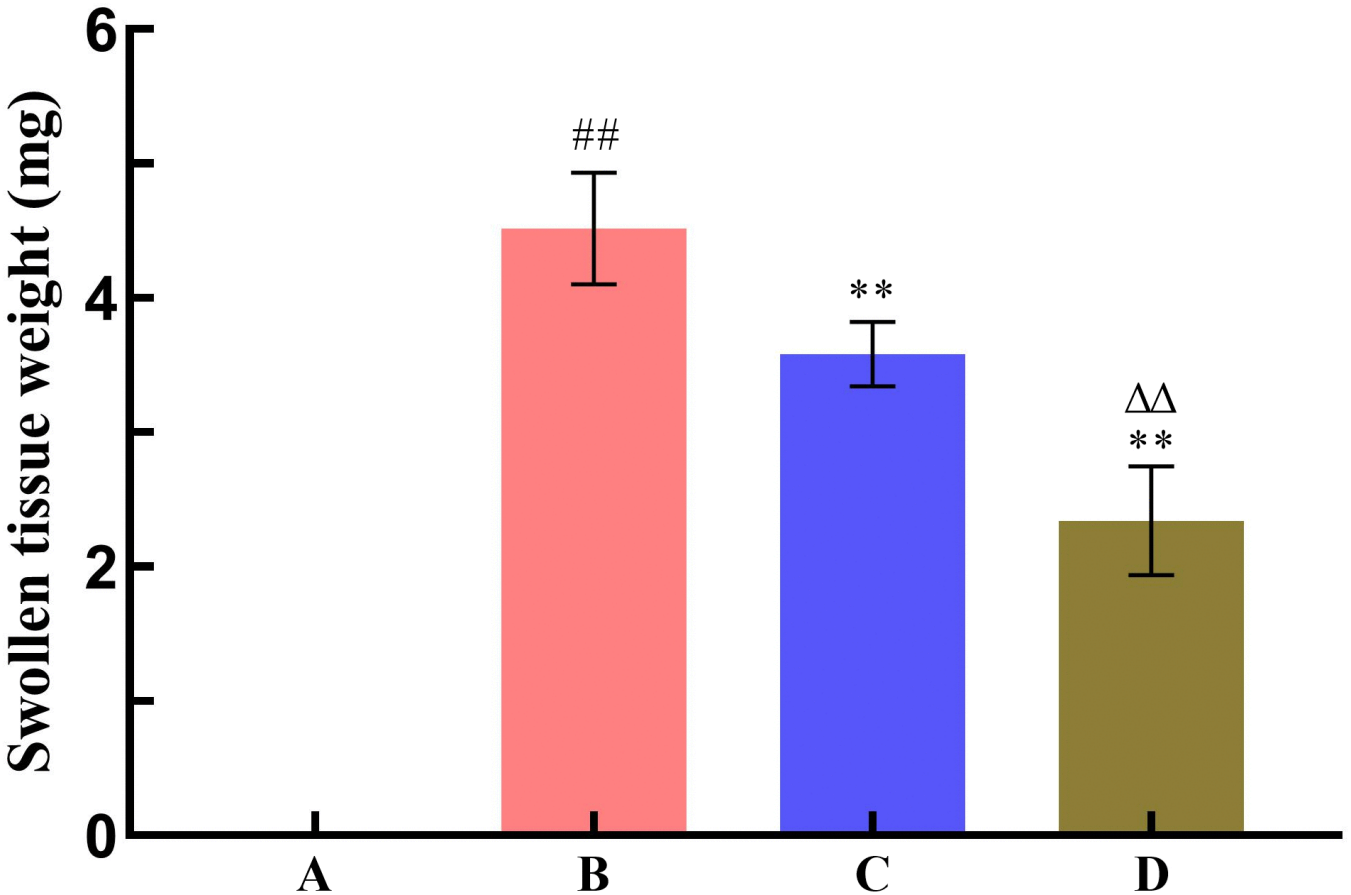
Effect of Radix Scutellariae on ear swelling in mice. Data are presented as *x̅* ± *s* (*n* = 10, error bars: SD). Significance: Compared with blank group (^#^: *P* < 0.05, ^##^: *P* < 0.01); Compared with model group (*: *P* < 0.05, **: *P* < 0.01); Compared with untreated Radix Scutellariae group (^Δ^: *P* < 0.05, ^ΔΔ^: *P* < 0.01). Groups: A (Blank Group), B (Model Group), C (untreated Radix Scutellariae Group), D (SNP-treated Radix Scutellariae Group). Swollen tissue weight: mg.

**Fig 11:**
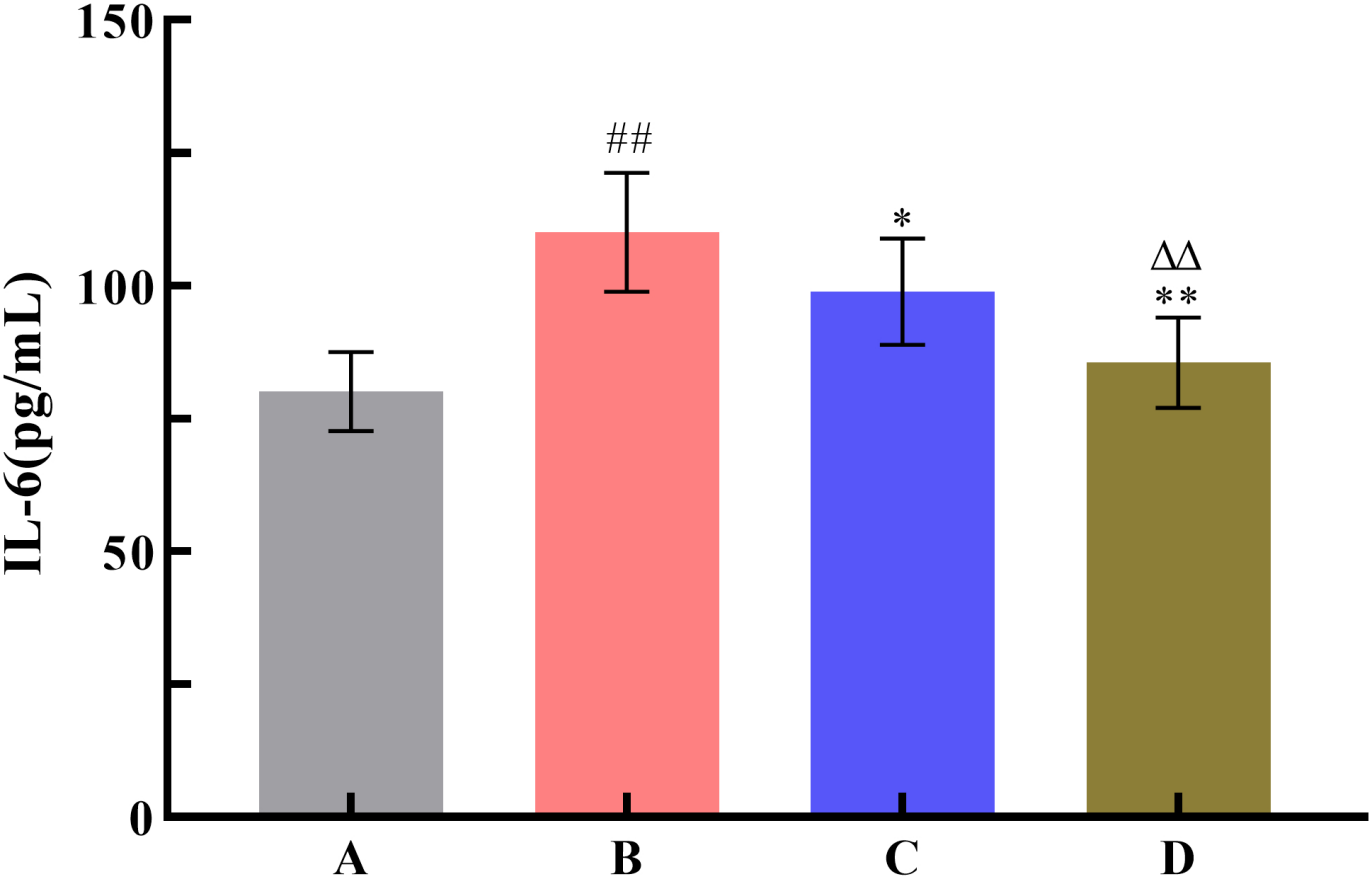
Effect of Radix Scutellariae on IL-6 in serum of mice. Data are presented as *x̅* ± *s* (*n* = 10, error bars: SD). Significance: Compared with blank group (^#^: *P* < 0.05, ^##^: *P* < 0.01); Compared with model group (*: *P* < 0.05, **: *P* < 0.01); Compared with untreated Radix Scutellariae group (^Δ^: *P* < 0.05, ^ΔΔ^: *P* < 0.01). Groups: A (Blank Group), B (Model Group), C (untreated Radix Scutellariae Group), D (SNP-treated Radix Scutellariae Group). IL-6 contents: pg/mL.

**Fig 12:**
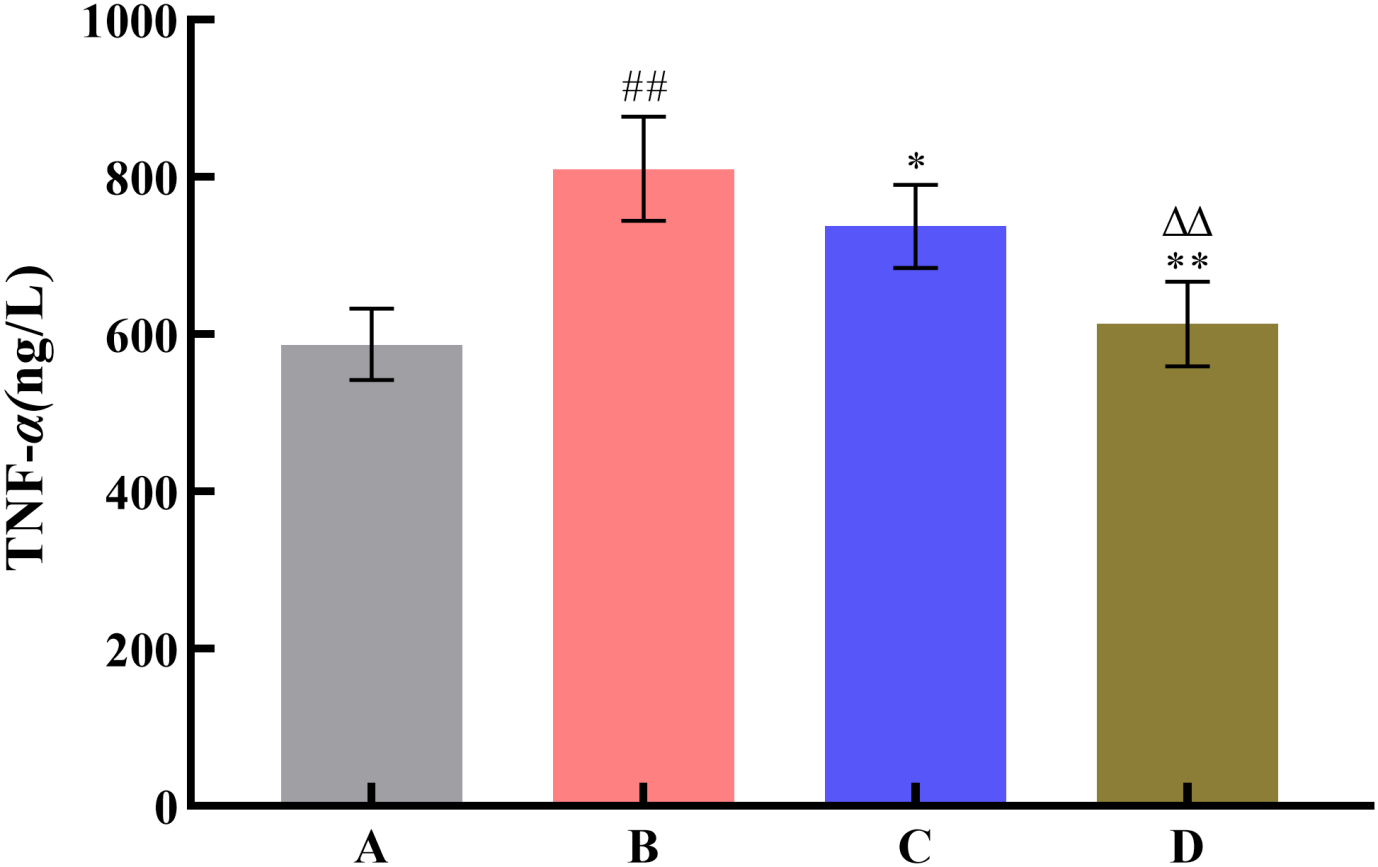
Effect of Radix Scutellariae on TNF-*α* in serum of mice. Data are presented as *x̅* ± *s* (*n* = 10, error bars: SD). Significance: Compared with blank group (^#^: *P* < 0.05, ^##^: *P* < 0.01); Compared with model group (*: *P* < 0.05, **: *P* < 0.01); Compared with untreated Radix Scutellariae group (^Δ^: *P* < 0.05, ^ΔΔ^: *P* < 0.01). Groups: A (Blank Group), B (Model Group), C (untreated Radix Scutellariae Group), D (SNP-treated Radix Scutellariae Group). TNF-*α* contents: ng/L.

To investigate the effect of SNP treatment on the pharmacodynamic activity of Radix Scutellariae, this study used the yeast-induced fever model to evaluate antipyretic activity, the acetic acid-induced writhing test to assess analgesic activity, and the xylene-induced ear edema model to examine anti-inflammatory activity. Compared with the model group, all Radix Scutellariae groups showed good efficacy. For antipyretic effects, the reduction in anal temperature was greater in the SNP-treated Radix Scutellariae group than in the untreated Radix Scutellariae group, with an increased inhibition rate of anal temperature elevation by 11.7%. For analgesic effects, the SNP-treated Radix Scutellariae group had better analgesic effects than the untreated group, with an average 4.2-fold reduction in the number of writhing responses and a 14.9% (*P* < 0.01) increase in the writhing inhibition rate. For anti-inflammatory effects, the SNP-treated group also performed best, with an average reduction in swollen tissue weight by 1.24 mg and an increase in the swelling inhibition rate by 27.4% (*P* < 0.01). Regarding the inflammatory factors IL-6 and TNF-*α*, the reduction in the SNP-treated Radix Scutellariae group was significantly higher than that in the untreated group, with reductions of 13.5% (*P* < 0.01) and 16.8% (*P* < 0.01), respectively (Table 1, Figs 8–12).

**Table 1:** Effect of SNP treatment on efficacy of Radix Scutellariae.

Groups	Temperature difference (°C)	Twisting times(n)	Swollen tissue weight (mg)	Inhibition rate of temperature elevation (%)	Writhing inhibition rate (%)	Swelling inhibition rate (%)	IL-6 content (pg/mL)	TNF-*α* content (ng/L)
A	0.22 ± 0.07	–	–	–	–	–	80.1 ± 7.5	587.2 ± 45.3
B	1.88 ± 0.32^##^	28.2 ± 4.4^##^	4.52 ± 0.42^##^	–	–	–	110.1 ± 11.2^##^	810.8 ± 66.2^##^
C	1.51 ± 0.39^*^	19.6 ± 2.7^**^	3.58 ± 0.24^**^	19.7	30.5	20.7	98.8 ± 10.0^*^	737.6 ± 53.0^*^
D	1.29 ± 0.30^**^	15.4 ± 2.2^**ΔΔ^	2.34 ± 0.40^**ΔΔ^	31.4	45.4	48.1	85.5 ± 8.5^**ΔΔ^	613.4 ± 54.0^**ΔΔ^

**Note: Data are presented as** $\overset{―}{x}\pm s$ **(*n* = 10, error bars: SD). Significance: Compared with blank group (**^**#**^**: *P* < 0.05,** ^**##**^: *P* < 0.01); Compared with model group (*: *P* < 0.05, **: *P* < 0.01); Compared with untreated Radix Scutellariae group (^**Δ**^**: *P* < 0.05,** ^**ΔΔ**^**: *P* < 0.01). Groups: A (Blank Group), B (Model Group), C (untreated Radix Scutellariae Group), D (SNP-treated Radix Scutellariae Group). “-” indicates no result.**

## Discussion

The biological nature of ecological stresses doing damage to plants is ROS. When plants are subjected to stress, ROS activate plants’ defense systems to protect the organisms from damage by altering enzyme activities, metabolism and osmotic balance, etc. [23]. The secondary metabolites are not only one of the main substances to eliminate ROS or to avoid ROS damage, but also the medicinal components, so the quality of herbal medicines is closely related to the secondary metabolism of plants, and the process of quality formation of herbal medicines is the process of physiological changes for plants adapting to environmental stress.

### Effect of SNP on ROS and MDA contents

ROS is one of the metabolites in the normal life activities of plants. The ROS levels are usually maintained in a relatively constant state by regulating their generation and elimination, but under environmental stresses a large number of ROS were produced and accumulated, and did damage to the biomolecules of life, leading to the lipid peroxidation of cytosolic membranes, with a result of a large amount of MDA being produced, so the MDA is used as an indicator to detect the degree of cellular damage [24]. Exogenous NO can induce the production of ROS and promote the accumulation of ROS [25]. Figs 2 and 3 show that the O_2_· ⁻ contents increased rapidly in *S*. *baicalensis* Georgi fresh roots, and the H_2_O_2_ contents began to accumulate, leading to MDA contents being also significantly increased. Their contents were positively correlated with the dose of SNP, indicating that the physiological response of plants under environmental stress could be constructed by exogenous NO. NO was in general consistent with that of O_2_·⁻ and H_2_O_2_, indicating that NO acted as a signaling molecule to initiate the production of ROS. However, the levels of NO, O_2_· ⁻ , and H_2_O_2_ not only did not continue to increase with increasing treatment time, but also decreased after day 3, probably because intracellular O_2_· ⁻ was mainly produced by xanthine oxidase and NADPH oxidase, and H_2_O_2_ was produced by SOD, xanthine oxidase, and protein kinase [9], and a large amount of ROS had a greater destructive effect on the enzyme structure [26], which ultimately led to the reduced ROS levels. In addition, NO has both oxidizing and reducing properties [27], and NO converts highly reactive O_2_·⁻ into the low-toxicity Peroxynitrite anion, which not only reduces ROS levels, but also mitigates damage to cell membranes [19]. The sharp increase in NO significantly increased ROS levels, which rapidly increased baicalein and wogonin contents, and ROS was eliminated and maintained at the original level, as shown in Fig 7.

### Effects of exogenous NO on antioxidant enzyme activities

Plants maintaining normal physiological metabolism must timely remove excess ROS by antioxidant substances [28], which is a unique ability formed during long-term evolution. Antioxidant enzymes, including SOD, CAT, POD and other antioxidant enzymes, are the main substances to eliminate ROS [29]. O_2_· ⁻ is the first ROS produced under environmental stress, and SOD as the first line of defense catalyzes the dismutation of O_2_·⁻ to O_2_ and H_2_O_2_ [30]. Next, H_2_O_2_ is reduced to nontoxic H_2_O by CAT, POD, etc. As an enzyme to specifically scavenge H_2_O_2_, CAT does not require additional reduced substrate; its activity is highly correlated with environmental stress [31]. POD requires polyphenols, polyamines or other substances as substrates, while polyphenols or polyamines are widespread secondary metabolites in plants and heavily synthesized and accumulated under stress conditions; therefore, POD also plays an important role in the adaptation of plants to adversity. More importantly, POD contains a large number of sugar groups, thus it is more stable; it also responds rapidly to a changing environment, so it is also known as the adversity enzyme [32], and it is more prominent under severe stress, Fig 4 also shows that POD can maintain higher stability in a higher ROS environment. CAT and POD are both enzymes that eliminate H_2_O_2_, and they synergize to convert the overproduced H_2_O_2_ into O_2_ and H_2_O, and protect cells from oxidative damage [29].

Antioxidant enzymes are also proteins, requiring chemical bonds such as hydrogen and disulfide bonds to maintain their corresponding secondary and tertiary structures to function, so ROS can regulate the function of proteins through the formation and cleavage of disulfide bonds [26]. In addition, ROS can bind to heme iron in antioxidant enzymes such as SOD, CAT, and POD to regulate O_2_·⁻ and H_2_O_2_ levels [33,34]. In the presence of SNPs, ROS are synthesized and accumulated in large quantities, and SOD as an inducible enzyme is enhanced by O_2_·⁻ under environmental stress [35]. Fig 4 shows that SOD, CAT and POD in the 7.5 and 20 mmol/L SNP-treated groups were significantly increased from 0 to 2 days, enhancing the elimination of O_2_· ⁻ , leading to a gradual decrease of O_2_· ⁻ levels from 2 to 6 days. CAT and POD synergistically complement each other in response to environmental stress. When one enzyme activity decreases, the other enzyme activity tends to increase to ensure the removal of ROS [36]. The CAT and POD activities of the 7.5 and 20 mmol/L SNP-treated groups both reached a peak on days 3 and 4, respectively, which synergistically reduced H_2_O_2_. Because POD has a strong resistance to ROS [32], it can still maintain high enzyme activity in the late stage, as shown in Fig.4, and plays a major role in the removal of H_2_O_2_. However, CAT activities were elevated only on day 3, probably due to the excessive ROS’s damaging effect. Fig 3 shows that the MDA contents had been maintained at a high level, meaning that the reduced antioxidant enzyme activities are due to ROS. The above indicates that exogenous NO can increase antioxidant enzymes’ activities and enhance the elimination of ROS, leading to reduced ROS after days 2–3. However, with the increase of stress or time, the activities of each antioxidant enzyme gradually decreased, indicating that antioxidant enzymes are not the main force to scavenge ROS under severe stress conditions.

### Effects of exogenous NO on secondary metabolite synthesis

Plants cannot move and often face various adversities, so they produce more ROS. Too much ROS will destroy proteins, including enzymes, so it is difficult for plants to eliminate too much ROS only by antioxidant enzymes. Why plants can survive is the evolution of secondary metabolic systems that animals do not have, by which the adaptability of plants to the external environment is improved [37]. As an important class of antioxidant substances, the secondary metabolites can either directly scavenge ROS or act as substrates for enzymes to scavenge ROS [38]; secondary metabolites are also induced by the environment or ROS, and ROS is the essential cause of the increase in secondary metabolism. Therefore, the relationship among ROS, key rate-limiting enzymes, and secondary metabolites is shown as follows: ROS↑→ key rate-limiting enzymes↑→ secondary metabolites↑→ ROS ↓ . This is a chain reaction of negative feedback; ROS originated from adversity stress is the root [39]. Flavonoids are a major class of secondary metabolites in plants, and their mechanisms of resistance to adversity are as follows: Firstly, they can absorb ultraviolet and reduce the damaging effects on macromolecules such as nucleic acids and proteins [40]. Secondly, flavonoids belong to polyphenolic compounds and can scavenge ROS [41]. The main secondary metabolites of *S*. *baicalensis* Georgi are exactly the flavonoids, including baicalin, wogonoside, baicalein, wogonin, and so on [42]. PAL, induced by environmental factors and signaling molecules such as ROS, is a key and rate-limiting enzyme for the biosynthesis of flavonoids [43]. Numerous studies have shown that when plants are subjected to stress, ROS burst, and the expression of key enzyme genes for secondary metabolism was enhanced. Fig 6 shows that exogenous NO can positively elevate the activities of PAL, and the flavonoid contents increase (Fig 7). The 20 mmol/L group exhibited the highest contents of baicalein and wogonin, attributed to high ROS accumulation, high PAL activity, and the sustained supply of 1,3-DPG (a raw material for secondary metabolite synthesis) (Figs 5, 6). In contrast, the 7.5 mmol/L group showed weaker stress intensity than the 20 mmol/L group. Although other indicators displayed similar trends under both concentrations, the lower ROS accumulation was insufficient to fully activate the secondary metabolic system of *S. baicalensis* Georgi; additionally, this group had the highest POD activity, which consumed a large amount of flavonoids during ROS scavenging [32], ultimately leading to the accumulation of baicalein and wogonin failing to increase as in the 20 mmol/L group, thus exhibiting a significant difference.

Not only are the flavonoids of *S*. *baicalensis* Georgi diverse, but also their activities vary considerably due to the differences in the number and position of -OH or -OCH_3_ groups. More importantly, their content and ratio change with the environmental conditions [22]. Therefore, it is difficult to assess the ecological effects of secondary metabolites with one or several flavones. Free flavonoids such as baicalein and wogonin not only contain more highly active -OH, but also diffuse easily to any parts of the cell to function due to the lack of water-soluble sugar groups. Free flavonoids have a very high bioactivity; it has been proven that the quality of Radix Scutellariae varies greatly among different origins [44]; the baicalin and wogonoside in Radix Scutellariae from the best producing area are not high, but the baicalein and wogonin are very high [45], so they are regarded as the characteristic chemical constituents of *S*. *baicalensis* Georgi subjected to adversity stress [46]. The flavonoids that SNP increased were mainly baicalein and wogonin, all free flavonoids, increasing by 1603.2% and 1256.1%, respectively, while the amounts of baicalin and wogonoside vary very little, which indicates that exogenous NO can significantly improve the quality of Radix Scutellariae. The latter decrease of ROS levels and the activities of antioxidant enzymes, the increase of baicalein and wogonin, also indicate that secondary metabolites play a major role in eliminating ROS under severe stress; ROS approaching the original level also means that the dynamic equilibrium of the new ROS is already established by regulating metabolism.

Phenylalanine is an intermediate product linking primary and secondary metabolism, and the enhanced activity of PAL (Fig 6) indicates that more primary metabolites are used for the biosynthesis of secondary metabolites. In this study, we used the fresh roots of *S*. *baicalensis* Georgi; the 1,3-DPG could not come from photosynthesis, but rather is the product of glucose glycolysis, relying on sugar substances to provide more energy and substances for secondary metabolism, so it can be inferred that the increased baicalein and wogonin are not converted from other flavonoids, but come from biosynthesis, which ensures the improvement of the herb’s quality.

### Effect of exogenous NO on the efficacy of Radix Scutellariae

The active ingredients of phytomedicines are complex; pharmacology is the most effective method to evaluate the quality. Radix Scutellariae has antipyretic, analgesic and anti-inflammatory effects. Table 1 shows that the SNP-treated group has a 11.7% increase in the inhibition rate of anal temperature, 14.9% in the torsion inhibition rate, 27.4% in the auricular swelling inhibition rate, and a 13.5% and 16.8% decrease in the serum levels of IL-6 and TNF-*α*, respectively, compared with those of the untreated group, indicating that SNP can significantly improve the efficacy of Radix Scutellariae. The main active ingredients of Radix Scutellariae include free flavonoids such as baicalein and wogonin, and flavonoid glycosides such as baicalin and wogonoside. Free flavonoids can be absorbed rapidly by passive diffusion through the mesenteric membrane due to their high lipid solubility and hydrophobicity, and flavonoid glycosides not only have a low lipid solubility but also have a high molecular weight and can only be absorbed after being converted to free flavonoids by intestinal flora [47,48], so the absorption rate of baicalein was about 7 times that of baicalin, and the efficacy was 4–7 times as great as baicalin [49,50]. Furthermore, baicalein exhibits superior pharmacological properties in a range of diseases, such as neurodegenerative diseases like Parkinson’s disease, acute liver injury, type 2 diabetes mellitus, myocardial fibrosis, and acute sepsis [51]. Therefore, the increased baicalein and wogonin in Radix Scutellariae are crucial to improve clinical efficacy. In this efficacy test, the baicalein and wogonin contents of the 20 mmol/L SNP group increased by 1603.2% and 1256.1%, respectively, which led to the enhancement of their medicinal efficacy.

### Considerations on the current study

This study was designed by artificially constructing the physiological state of plants under stress. The selected plant samples may affect the efficacy of SNP treatment due to differences in ROS levels; additionally, this study only focused on a single batch of *S. baicalensis* Georgi, with a limited number of experimental samples. Secondly, the purpose of this study is to clarify the formation mechanism of medicinal material quality, focusing on identifying the macroscopic changes in the stress physiological state of plants. It only focuses on physiological state analysis, lacking systematic research on gene transcriptional regulation, key gene expression data, and secondary metabolic pathways, thus having deficiencies in mechanism exploration. However, this study confirms the feasibility of artificial intervention in secondary metabolism, which can provide directions for subsequent research on the formation mechanism of medicinal material quality carried out through metabolomics and gene expression analysis, systematic analysis of related molecular pathways, and other approaches.

## Conclusion

Exogenous NO can increase ROS levels in the fresh roots of *S*. *baicalensis* Georgi, and can replace adversity, improve the activities of antioxidant enzymes and the contents of their secondary metabolites; they worked together to eliminate excessive ROS and reduce the damage caused by ROS to the organism. Under severe stress conditions, the activities of antioxidant enzymes were weakened, and secondary metabolism played a leading role; the baicalein and wogonin with high biological activity increased remarkably, and the antipyretic, analgesic, and anti-inflammatory effects were significantly enhanced. Therefore, ROS burst could be induced in the fresh roots of *S*. *baicalensis* Georgi by exogenous NO; SNP could improve the herb quality of cultivated Radix Scutellariae.

## Supporting information

S1 File Original data. (ZIP)

## References

[1] Shen J, Li P, Liu S, Liu Q, Li Y, Sun Y, et al. Traditional uses, ten-years research progress on phytochemistry and pharmacology, and clinical studies of the genus Scutellaria. J Ethnopharmacol. 2021;265:113198. doi: 10.1016/j.jep.2020.113198 32739568

[2] Zhao T, Tang H, Xie L, Zheng Y, Ma Z, Sun Q, et al. Scutellaria baicalensis Georgi. (Lamiaceae): a review of its traditional uses, botany, phytochemistry, pharmacology and toxicology. J Pharm Pharmacol. 2019;71(9):1353–69. doi: 10.1111/jphp.13129 31236960

[3] Li C, Lin G, Zuo Z. Pharmacological effects and pharmacokinetics properties of Radix Scutellariae and its bioactive flavones. Biopharm Drug Dispos. 2011;32(8):427–45. doi: 10.1002/bdd.771 21928297

[4] Dai YJ, Wan SY, Gong SS, Liu JC, Li F, Kou JP. Recent advances of traditional Chinese medicine on the prevention and treatment of COVID-19. Chin J Nat Med. 2020;18(12):881–9. doi: 10.1016/S1875-5364(20)60031-0 33357718 PMC7832371

[5] Zhang C, Zhong X, Bao S, Urtnasan M, Li M. Main factors affecting the efficacy of medicinal plants during the cultivation process. Front Plant Sci. 2025;16:1634926. doi: 10.3389/fpls.2025.1634926 41036395 PMC12479407

[6] Jangpangi D, Patni B, Chandola V, Chandra S. Medicinal plants in a changing climate: understanding the links between environmental stress and secondary metabolite synthesis. Front Plant Sci. 2025;16:1587337. doi: 10.3389/fpls.2025.1587337 40584865 PMC12202592

[7] Singh AK, Dhanapal S, Yadav BS. The dynamic responses of plant physiology and metabolism during environmental stress progression. Mol Biol Rep. 2020;47(2):1459–70. doi: 10.1007/s11033-019-05198-4 31823123

[8] Choudhury S, Panda P, Sahoo L, Panda SK. Reactive oxygen species signaling in plants under abiotic stress. Plant Signal Behav. 2013;8(4):e23681. doi: 10.4161/psb.23681 23425848 PMC7030282

[9] Wang P, Liu W-C, Han C, Wang S, Bai M-Y, Song C-P. Reactive oxygen species: Multidimensional regulators of plant adaptation to abiotic stress and development. J Integr Plant Biol. 2024;66(3):330–67. doi: 10.1111/jipb.13601 38116735

[10] Dat J, Vandenabeele S, Vranová E, Van Montagu M, Inzé D, Van Breusegem F. Dual action of the active oxygen species during plant stress responses. Cell Mol Life Sci. 2000;57(5):779–95. doi: 10.1007/s000180050041 10892343 PMC11147059

[11] You J, Chan Z. ROS Regulation During Abiotic Stress Responses in Crop Plants. Front Plant Sci. 2015;6:1092. doi: 10.3389/fpls.2015.01092 26697045 PMC4672674

[12] Czarnocka W, Karpiński S. Friend or foe? Reactive oxygen species production, scavenging and signaling in plant response to environmental stresses. Free Radic Biol Med. 2018;122:4–20. doi: 10.1016/j.freeradbiomed.2018.01.011 29331649

[13] Considine MJ, Sandalio LM, Foyer CH. Unravelling how plants benefit from ROS and NO reactions, while resisting oxidative stress. Ann Bot. 2015;116(4):469–73. doi: 10.1093/aob/mcv153 26649372 PMC4578007

[14] Forman HJ, Maiorino M, Ursini F. Signaling functions of reactive oxygen species. Biochemistry. 2010;49(5):835–42. doi: 10.1021/bi9020378 20050630 PMC4226395

[15] Huimin G, Xiaoying F, Hongwei D, Wei C, Xiangcai M. Sodium dithionite-enhanced quality of radix scutellariae through modification of secondary metabolism. Int J Pharm Investig. 2016;6(4):225–30. doi: 10.4103/2230-973X.195932 28123992 PMC5204254

[16] Ramakrishna Rao DN, Cederbaum AI. Generation of reactive oxygen species by the redox cycling of nitroprusside. Biochim Biophys Acta. 1996;1289(2):195–202. doi: 10.1016/0304-4165(95)00158-1 8600973

[17] Wu J, Zhang Y, Hao R, Cao Y, Shan X, Jing Y. Nitric Oxide Enhances Cytotoxicity of Lead by Modulating the Generation of Reactive Oxygen Species and Is Involved in the Regulation of Pb2+ and Ca2+ Fluxes in Tobacco BY-2 Cells. Plants (Basel). 2019;8(10):403. doi: 10.3390/plants8100403 31600951 PMC6843202

[18] Koppenol WH. The basic chemistry of nitrogen monoxide and peroxynitrite. Free Radic Biol Med. 1998;25(4–5):385–91. doi: 10.1016/s0891-5849(98)00093-8 9741577

[19] Corpas FJ, González-Gordo S, Palma JM. Nitric oxide and hydrogen sulfide modulate the NADPH-generating enzymatic system in higher plants. J Exp Bot. 2021;72(3):830–47. doi: 10.1093/jxb/eraa440 32945878

[20] Sudha G, Ravishankar GA. Involvement and interaction of various signaling compounds on the plant metabolic events during defense response, resistance to stress factors, formation of secondary metabolites and their molecular aspects. Plant Cell Tiss Org Cult. 2002;71(3):181–212. doi: 10.1023/a:1020336626361

[21] Ge Y, Chen Y, Li C, Zhao J, Wei M, Li X, et al. Effect of sodium nitroprusside treatment on shikimate and phenylpropanoid pathways of apple fruit. Food Chem. 2019;290:263–9. doi: 10.1016/j.foodchem.2019.04.010 31000046

[22] Shen Y, Cong W, Zhang A-H, Meng X. Complexity of active medicinal ingredients in radix scutellariae with sodium hydrosulfite exposure. PLoS One. 2020;15(9):e0238927. doi: 10.1371/journal.pone.0238927 32956425 PMC7505437

[23] Hossain MA, Bhattacharjee S, Armin S-M, Qian P, Xin W, Li H-Y, et al. Hydrogen peroxide priming modulates abiotic oxidative stress tolerance: insights from ROS detoxification and scavenging. Front Plant Sci. 2015;6:420. doi: 10.3389/fpls.2015.00420 26136756 PMC4468828

[24] Wang B, Wang Y, Zhang J, Hu C, Jiang J, Li Y, et al. ROS-induced lipid peroxidation modulates cell death outcome: mechanisms behind apoptosis, autophagy, and ferroptosis. Arch Toxicol. 2023;97(6):1439–51. doi: 10.1007/s00204-023-03476-6 37127681

[25] Corpas FJ, Río LAD, Palma JM. Impact of Nitric Oxide (NO) on the ROS Metabolism of Peroxisomes. Plants (Basel). 2019;8(2):37. doi: 10.3390/plants8020037 30744153 PMC6409570

[26] Ye J, Bazzi S, Fritz T, Tittmann K, Mata RA, Uranga J. Mechanisms of Cysteine-Lysine Covalent Linkage-The Role of Reactive Oxygen Species and Competition with Disulfide Bonds. Angew Chem Int Ed Engl. 2023;62(36):e202304163. doi: 10.1002/anie.202304163 37294559

[27] Khan M, Al Azzawi TNI, Ali S, Yun B-W, Mun B-G. Nitric Oxide, a Key Modulator in the Alleviation of Environmental Stress-Mediated Damage in Crop Plants: A Meta-Analysis. Plants (Basel). 2023;12(11):2121. doi: 10.3390/plants12112121 37299100 PMC10255435

[28] Baxter A, Mittler R, Suzuki N. ROS as key players in plant stress signalling. J Exp Bot. 2014;65(5):1229–40. doi: 10.1093/jxb/ert375 24253197

[29] Rajput VD, Harish, Singh RK, Verma KK, Sharma L, Quiroz-Figueroa FR, et al. Recent Developments in Enzymatic Antioxidant Defence Mechanism in Plants with Special Reference to Abiotic Stress. Biology (Basel). 2021;10(4):267. doi: 10.3390/biology10040267 33810535 PMC8066271

[30] Gill SS, Anjum NA, Gill R, Yadav S, Hasanuzzaman M, Fujita M, et al. Superoxide dismutase--mentor of abiotic stress tolerance in crop plants. Environ Sci Pollut Res Int. 2015;22(14):10375–94. doi: 10.1007/s11356-015-4532-5 25921757

[31] Yang T, Poovaiah BW. Hydrogen peroxide homeostasis: activation of plant catalase by calcium/calmodulin. Proc Natl Acad Sci U S A. 2002;99(6):4097–102. doi: 10.1073/pnas.052564899 11891305 PMC122654

[32] Czégény G, Rácz A. Phenolic peroxidases: Dull generalists or purposeful specialists in stress responses? J Plant Physiol. 2023;280:153884. doi: 10.1016/j.jplph.2022.153884 36543063

[33] Clark D, Durner J, Navarre DA, Klessig DF. Nitric oxide inhibition of tobacco catalase and ascorbate peroxidase. Mol Plant Microbe Interact. 2000;13(12):1380–4. doi: 10.1094/MPMI.2000.13.12.1380 11106031

[34] Klessig DF, Durner J, Noad R, Navarre DA, Wendehenne D, Kumar D, et al. Nitric oxide and salicylic acid signaling in plant defense. Proc Natl Acad Sci U S A. 2000;97(16):8849–55. doi: 10.1073/pnas.97.16.8849 10922045 PMC34022

[35] Wang W, Xia MX, Chen J, Yuan R, Deng FN, Shen FF. Gene Expression Characteristics and Regulation Mechanisms of Superoxide Dismutase and Its Physiological Roles in Plants under Stress. Biochemistry (Mosc). 2016;81(5):465–80. doi: 10.1134/S0006297916050047 27297897

[36] Dvořák P, Krasylenko Y, Zeiner A, Šamaj J, Takáč T. Signaling Toward Reactive Oxygen Species-Scavenging Enzymes in Plants. Front Plant Sci. 2021;11:618835. doi: 10.3389/fpls.2020.618835 33597960 PMC7882706

[37] Pant P, Pandey S, Dall’Acqua S. The Influence of Environmental Conditions on Secondary Metabolites in Medicinal Plants: A Literature Review. Chem Biodivers. 2021;18(11):e2100345. doi: 10.1002/cbdv.202100345 34533273

[38] Avasthi A, Kaur N, Sharda S, Ghosal S. An Insight into Antioxidant and Antimicrobial Activities of Ethnotherapeutically Important Trans Himalayan Medicinal Plants: A Review. J Pharm Res Int. 2021;33(36A):195–212.

[39] Cao S, Shi L, Shen Y, He L, Meng X. Ecological roles of secondary metabolites of Saposhnikovia divaricata in adaptation to drought stress. PeerJ. 2022;10:e14336. doi: 10.7717/peerj.14336 36353606 PMC9639429

[40] Zhuang W-B, Li Y-H, Shu X-C, Pu Y-T, Wang X-J, Wang T, et al. The Classification, Molecular Structure and Biological Biosynthesis of Flavonoids, and Their Roles in Biotic and Abiotic Stresses. Molecules. 2023;28(8):3599. doi: 10.3390/molecules28083599 37110833 PMC10147097

[41] Yeshi K, Ruscher R, Miles K, Crayn D, Liddell M, Wangchuk P. Antioxidant and Anti-Inflammatory Activities of Endemic Plants of the Australian Wet Tropics. Plants (Basel). 2022;11(19):2519. doi: 10.3390/plants11192519 36235388 PMC9571949

[42] Lu T, Song J, Huang F, Deng Y, Xie L, Wang G, et al. Comparative pharmacokinetics of baicalin after oral administration of pure baicalin, Radix scutellariae extract and Huang-Lian-Jie-Du-Tang to rats. J Ethnopharmacol. 2007;110(3):412–8. doi: 10.1016/j.jep.2006.09.036 17110066

[43] Qi S, Wu-Lin C, Hua J, Ai-Hua Z, Xiang-Cai M. H_2_O_2_ Improves Quality of Radix scutellariae Through Anti-oxidant Effect. Pharmacogn Mag. 2016;12(45):84–90. doi: 10.4103/0973-1296.176063 27019566 PMC4787343

[44] Guo L, Wang S, Zhang J, Yang G, Zhao M, Ma W, et al. Effects of ecological factors on secondary metabolites and inorganic elements of *Scutellaria baicalensis* and analysis of geoherblism. Sci China Life Sci. 2013;56(11):1047–56. doi: 10.1007/s11427-013-4562-5 24203454

[45] Lv K, Yin C, Li F, Chen W, Zhao L, Liu Z, et al. Rapid and comprehensive quality evaluation of Huang-qin from different origins by FT-IR and NIR spectroscopy combined with chemometrics. Phytochem Anal. 2024;35(7):1587–99. doi: 10.1002/pca.3402 38850098

[46] Wang Z-L, Wang S, Kuang Y, Hu Z-M, Qiao X, Ye M. A comprehensive review on phytochemistry, pharmacology, and flavonoid biosynthesis of *Scutellaria baicalensis*. Pharm Biol. 2018;56(1):465–84. doi: 10.1080/13880209.2018.1492620 31070530 PMC6292351

[47] Taiming L, Xuehua J. Investigation of the absorption mechanisms of baicalin and baicalein in rats. J Pharm Sci. 2006;95(6):1326–33. doi: 10.1002/jps.20593 16628739

[48] Ganguly R, Gupta A, Pandey AK. Role of baicalin as a potential therapeutic agent in hepatobiliary and gastrointestinal disorders: A review. World J Gastroenterol. 2022;28(26):3047–62. doi: 10.3748/wjg.v28.i26.3047 36051349 PMC9331529

[49] Cai Y, Li S, Li T, Zhou R, Wai AT-S, Yan R. Oral pharmacokinetics of baicalin, wogonoside, oroxylin A 7-O-*β*-d-glucuronide and their aglycones from an aqueous extract of Scutellariae Radix in the rat. J Chromatogr B Analyt Technol Biomed Life Sci. 2016;1026:124–33. doi: 10.1016/j.jchromb.2015.11.049 26809374

[50] Lai M-Y, Hsiu S-L, Tsai S-Y, Hou Y-C, Chao P-DL. Comparison of metabolic pharmacokinetics of baicalin and baicalein in rats. J Pharm Pharmacol. 2003;55(2):205–9. doi: 10.1211/002235702522 12631413

[51] Hu Z, Guan Y, Hu W, Xu Z, Ishfaq M. An overview of pharmacological activities of baicalin and its aglycone baicalein: New insights into molecular mechanisms and signaling pathways. Iran J Basic Med Sci. 2022;25(1):14–26. doi: 10.22038/IJBMS.2022.60380.13381 35656442 PMC9118284

